# BRCA1 is a key regulator of breast differentiation through activation of Notch signalling with implications for anti-endocrine treatment of breast cancers

**DOI:** 10.1093/nar/gkt626

**Published:** 2013-07-17

**Authors:** Niamh E. Buckley, Caoimhe B. Nic An tSaoir, Jaine K. Blayney, Lisa C. Oram, Nyree T. Crawford, Zenobia C. D’Costa, Jennifer E. Quinn, Richard D. Kennedy, D. Paul Harkin, Paul B. Mullan

**Affiliations:** Centre for Cancer Research and Cell Biology, Queen’s University Belfast, 97 Lisburn Road, Belfast BT7 9BL, UK

## Abstract

Here, we show for the first time, that the familial breast/ovarian cancer susceptibility gene BRCA1 activates the Notch pathway in breast cells by transcriptional upregulation of Notch ligands and receptors in both normal and cancer cells. We demonstrate through chromatin immunoprecipitation assays that BRCA1 is localized to a conserved intronic enhancer region within the Notch ligand Jagged-1 (JAG1) gene, an event requiring ΔNp63. We propose that this BRCA1/ΔNp63-mediated induction of JAG1 may be important the regulation of breast stem/precursor cells, as knockdown of all three proteins resulted in increased tumoursphere growth and increased activity of stem cell markers such as Aldehyde Dehydrogenase 1 (ALDH1). Knockdown of Notch1 and JAG1 phenocopied BRCA1 knockdown resulting in the loss of Estrogen Receptor-α (ER-α) expression and other luminal markers. A Notch mimetic peptide could activate an ER-α promoter reporter in a BRCA1-dependent manner, whereas Notch inhibition using a γ-secretase inhibitor reversed this process. We demonstrate that inhibition of Notch signalling resulted in decreased sensitivity to the anti-estrogen drug Tamoxifen but increased expression of markers associated with basal-like breast cancer. Together, these findings suggest that BRCA1 transcriptional upregulation of Notch signalling is a key event in the normal differentiation process in breast tissue.

## INTRODUCTION

BRCA1 was the first identified breast and ovarian cancer susceptibility gene responsible for approximately half of all inherited breast cancer cases (1). Women who carry a BRCA1 germ line mutation have a cumulative lifetime risk of 50–85% of developing breast cancer (2). Somatic BRCA1 mutations are rare in sporadic breast cancer, but BRCA1 expression is downregulated in ∼30% of sporadic cases (3). BRCA1 is known to have multiple roles including DNA damage repair, cell cycle checkpoint control, ubiquitination and transcriptional regulation. Although BRCA1 does not bind to DNA in a sequence specific manner, it facilitates transcriptional control at a number of different levels through its ability to interact with proteins such as transcription factors, the RNA polymerase II holoenzyme complex and proteins involved in chromatin remodelling [for review see (4)]. Through these multiple interactions, BRCA1 can co-activate or co-repress a large number of target genes involved in its downstream functions.

The mammary gland comprises a branched network of ductal epithelial structures terminating in alveoli, composed of two distinct cell types, luminal (secretory) and basal (myoepithelial). BRCA1 deficient tumours exhibit characteristics similar to the basal-like subtype of breast tumours, which resemble the gene expression pattern of basal epithelial cells (5). These include ‘triple negative’ receptor status (low ER-α, Progesterone Receptor and HER2 expression), strong expression of basal cytokeratins, high p53 mutation rates, impaired differentiation and poor prognosis. BRCA1 expression has been shown to be required for the differentiation of ER-α-negative stem/progenitor cells to ER-α-positive luminal cells with abrogation of BRCA1 leading to increased stem cell activity (6). Our colleagues have found that BRCA1 may regulate luminal differentiation through its ability to transcriptionally activate ER-α (7). BRCA1 mutation carriers have been shown to have an expanded luminal progenitor population within the breast implying this subset may be most susceptible to BRCA1 dysfunction (8,9). When BRCA1 expression is abrogated specifically in the luminal progenitor subpopulation, mice develop mammary tumours that phenocopy human BRCA1 breast cancers (10).

The Notch pathway is a juxtacrine signalling pathway important for the normal functioning and development of multiple tissues. The canonical Notch pathway consists of four receptors (Notch 1–4) and five ligands [delta-like-1, -3 and -4 (DLL1, DLL3 and DLL4), Jagged1 and Jagged2 (JAG1 and JAG2)]. Notch ligands share a Delta-Serrate-Lag (DSL) region, which is critical for receptor recognition and activation. Notch ligand-receptor docking between two neighbouring cells is followed by two proteolytic cleavages of the respective Notch receptor (including cleavage by γ-secretase) to liberate the cytoplasmic part of the receptor called the Notch Intracellular Domain (NICD). The NICD translocates to the nucleus and recruits histone acetyltransferases to the transcription factor CBF-1/CSL/RBP-J*κ* to form a transcriptional activation complex on CSL-responsive promoters. Notch signalling is essential for mammary stem cell commitment to differentiation, and targeted deletion of Cbf-1 resulted in increased stem cell activity and aberrant mammary end-bud formation (11). Mice with *Notch1*-deficient epithelia develop spontaneous basal cell-like skin carcinomas (12), and cre-mediated loss of CSL led to an accumulation of basal cell clusters during pregnancy and excessive proliferation of basal-like cells in the mammary gland (13). Notch activation is known to be associated with the transition from bipotent mammary progenitors to luminally differentiated populations with Notch3 expression, in particular, being essential (14). Notch4 activation is associated with the basal layer and the proliferation of breast progenitor/stem cells, whereas Notch1, 2 and 3 are all associated with luminal subpopulations (11,15,16). The Notch pathway may, therefore, be a key regulator of mammary alveolar development during pregnancy by maintaining luminal cell fate and preventing uncontrolled basal cell proliferation.

In this study, we show that BRCA1 activates the Notch pathway in both non-tumorigenic and breast cancer cells, in addition to primary breast tumours, through transcriptional upregulation of Notch receptors and ligands. We find that BRCA1 regulates JAG1 gene in a ΔNp63-dependent mechanism. We show that BRCA1, ΔNp63 and JAG1 may all play roles in stem cell regulation, as knockdown of all three proteins result in increased ALDH1 activity and tumoursphere growth. Short interfering RNA (siRNA) knockdown of Notch signalling components in this pathway resulted in the loss of markers associated with basal and luminal differentiation, whereas proliferation-associated genes and markers of basal-like breast cancer were increased. We show that knockdown of Notch1 and JAG1 phenocopy the knockdown of BRCA1 resulting in the loss of ERα and luminal marker expression. Exogenous activation of Notch signalling resulted in increased activation of the ER-α promoter, whereas Notch inhibition by a γ-secretase inhibitor reversed this process. Consequently, inhibition of the Notch pathway led to decreased sensitivity to the anti-estrogen, Tamoxifen, and increased expression of markers associated with basal-like breast cancer. These findings show for the first time that BRCA1 may regulate mammary cell fate through transcriptional activation of the Notch pathway, ensuring normal basal and luminal differentiation in breast tissue. Furthermore, the use of Notch pathway inhibitors for the treatment of ER-α positive breast cancer may disrupt these signalling mechanisms resulting in the selection of more aggressive basal-like tumour subtypes.

## MATERIALS AND METHODS

### Cell lines

Cell lines were characterized by isoenzyme/cytochrome c oxidase I assay and short tandem repeat analysis by LGC Standards. Full details of the HCC-EV/BR, MCF-7, T47D and HME-1 cell lines are provided in (17). The 184A1 cells are a spontaneously immortalised cell line derived from reduction mammoplasty tissue and were a kind gift from Dr Martha Stampfer (University of California) and maintained as described in (18). shRNA cells lines were generated as previously described (19). γ-secretase (DAPT Calbiochem) was used at 1 μM. Treatment of cells with DSL peptide was carried out as previously described (15). Tamoxifen (Sigma) was used according to manufacturer’s instructions, and cell viability was assessed by MTT (Thiazolyl Blue Tetrazolium Bromide - Sigma). Tumoursphere cultures and Aldefluor assays were carried out as previously described (19) and outlined briefly in Supplementary Data.

### Murine embryonic stem cells

BRCA1 wild-type and BRCA1 null murine embryonic stem (ES) cells were a kind gift from Prof. Alan Ashworth (Institute of Cancer Research, London). ES cells were grown as previously described (20).

### Flow cytometry

Cells were stained with CD49f, EpCAM and CD24 along with relevant immunoglobulin G (IgG) controls according to manufacturer’s instructions (BD Biosciences). Data were analysed as described by Keller *et al.* (21).

### siRNA

siRNA transfection were carried out as previously described (22). The siRNA sequences are shown in Supplementary Data.

### Generation of luciferase constructs

The luciferase construct pGL3tkJ1IER was cloned as previously described (23). Notch 1 promoter region −264 to 228 was PCR amplified and cloned into pGL3 basic (pGL3N1). Primers are detailed in Supplementary Data.

### Luciferase reporter assays

Luciferase assays were carried out as previously described (7).

### Immunoblot analysis

Immunoblot analysis was performed as previously described (24). Primary antibodies are listed in Supplementary Data.

### Real-time quantitative PCR

Real-time quantitative PCR (RqPCR) was carried out as previously described (7). Primers are detailed in Supplementary Data.

### Chromatin immunoprecipitation assays

Chromatin immunoprecipitation (ChIP) assays performed as previously described (7). Primers used are shown in Supplementary Data.

### Gene expression analysis

Microarray profiles of an in-house data set and a publically available data set (GSE1456) were obtained (additional information in Supplementary Data). Samples were background-corrected, normalized and transformed using the Affy package, justRMA. Individual probe sets corresponding to genes of interest were identified. For each gene, a median value of expression intensity was calculated from the relevant probe sets. This median intensity was compared in the two groups, BRCA1 sporadic versus BRCA1 mutant using boxplots.

### Statistical analysis

All relevant data were analysed by two-tailed Students *t*-test except Supplementary Figure S7, which was analysed by Mann–Whitney Two-group Unpaired Test. All data were deemed significant with a *P*-value of at least <0.05. All *P*-values are included in Supplementary Data Set S1.

## RESULTS

### BRCA1 activates Notch signalling

**Figure 1.**
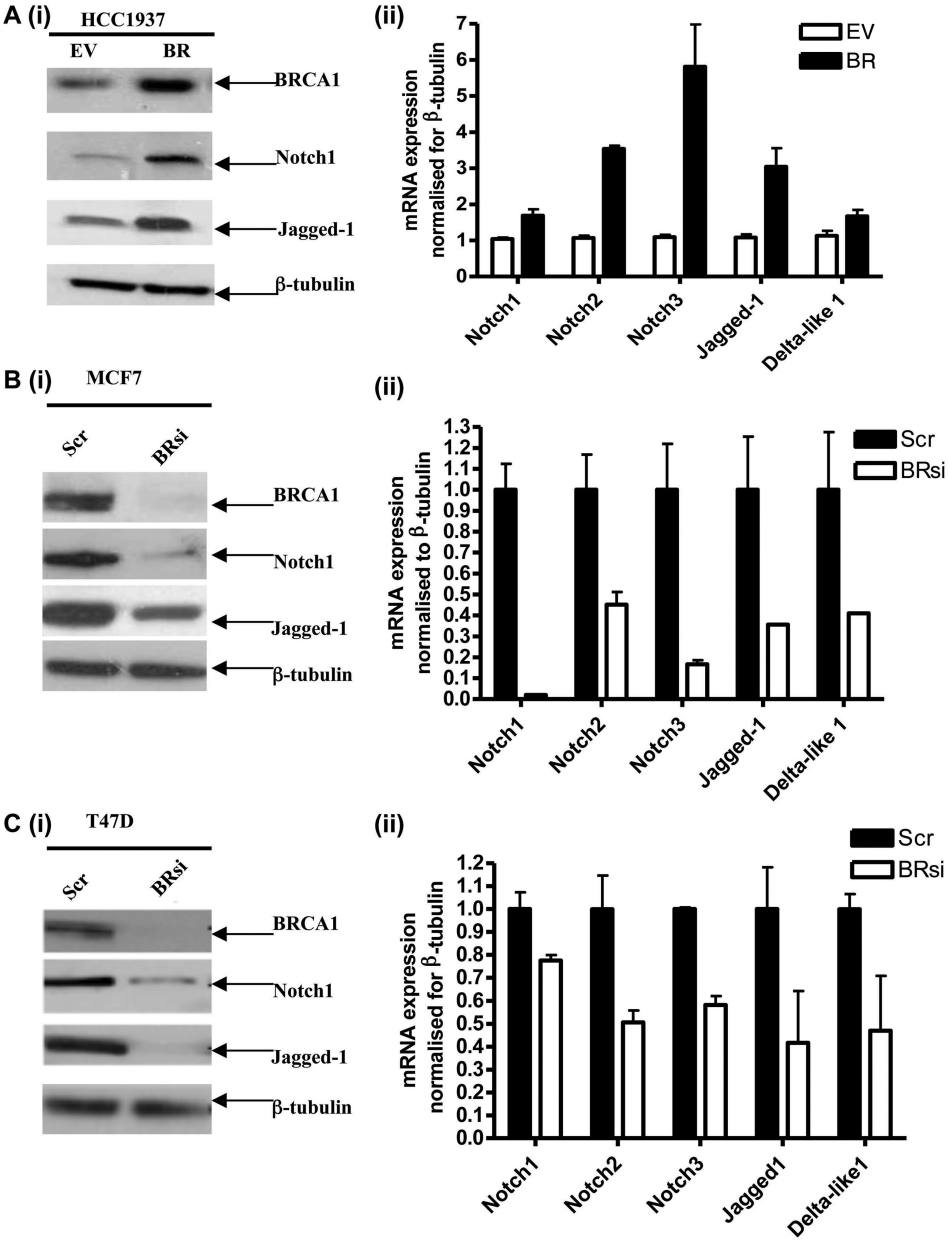
(**A**) (i) Immunoblots of HCC1937 cells stably transfected with an empty vector (EV) or an expression construct containing wild-type BRCA1 (BR). Blots were probed with the antibodies shown and reprobed with β-tubulin as a loading control. (ii) RqPCR of the same cell lines described in (i) with primers specific for Notch receptors and ligands. (**B**) (i) Immunoblot of MCF-7 cells following scrambled (Scr) control or BRCA1 (BRsi)-specific siRNA treatments. Blots were probed with the antibodies shown and reprobed with β-tubulin as a loading control. (ii) RqPCR of the cells described in (i) with primers specific for Notch receptors and ligands. (**C**) (i) Immunoblot of T47D cells following the siRNA treatments described for MCF-7 cells with (ii) the same corresponding RqPCR analyses.

**Figure 2.**
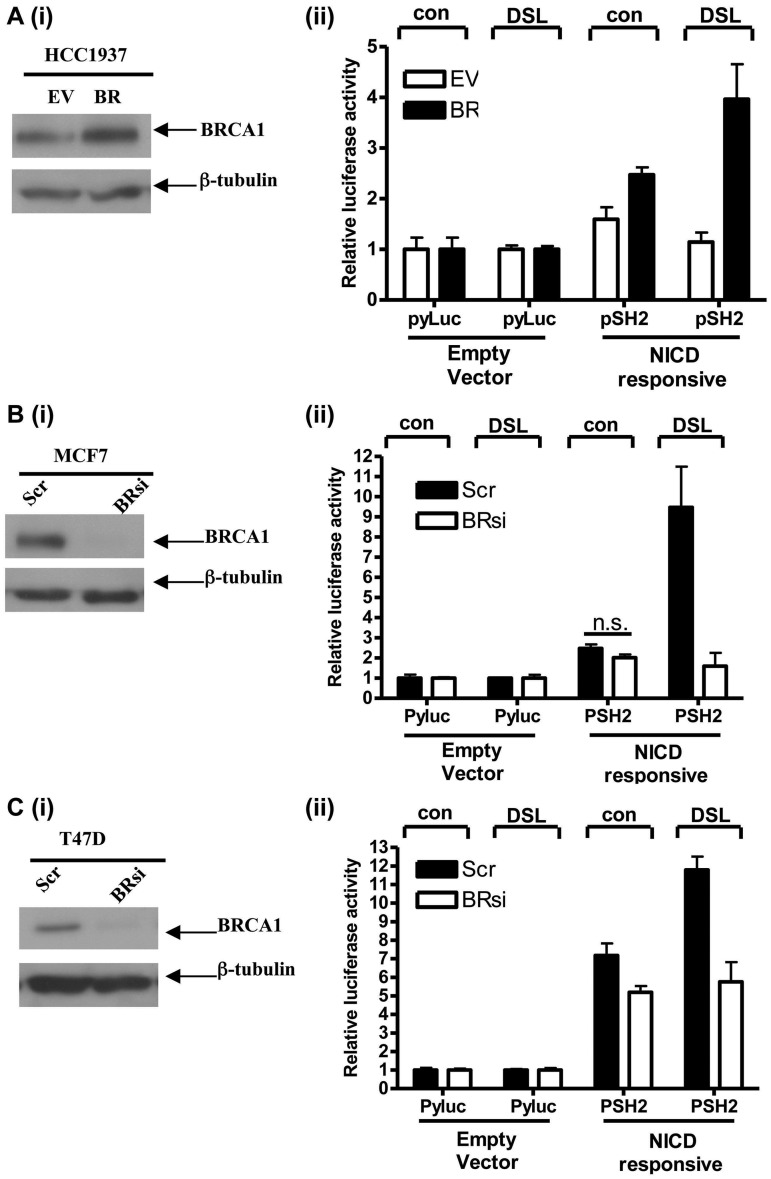
(**A**) (i) Immunoblot of HCC EV and BR cells probed for BRCA1 and reprobed with β-tubulin as a loading control. (ii) Luciferase reporter assay of HCC EV and BR cells following transfection of a Notch-responsive β-globin reporter construct (pSH2) and an empty vector control (pyLuc) with co-transfection of a Renilla luciferase construct used for transfection control. Cells were pretreated with a scrambled peptide (con) or a Notch ligand mimicking peptide (DSL). (**B**) (i) Immunoblot of MCF-7 cells following scrambled (Scr) control or BRCA1 (BRsi)-specific siRNA treatments. Blot was probed for BRCA1 and reprobed with β-tubulin as a loading control. (ii) Reporter assay was performed as described in (A) (ii). (**C**) (i) Immunoblot of T47D cells following the siRNA treatments described for MCF-7 cells with (ii) the reporter assay as described in (A) (ii).

Following microarray analysis, we identified several Notch pathway genes as BRCA1 upregulated targets following reconstitution of wild-type BRCA1 into BRCA1 mutant HCC1937 cells (25). We validated by western blotting [Figure 1A (i)] and by RqPCR [Figure 1A (ii)] that Notch ligands JAG1 and DLL-1 and receptors Notch1, 2, and 3 were all BRCA1 transcriptional targets. Conversely, siRNA knockdown of BRCA1 in BRCA1 wild-type MCF-7 (Figure 1B and Supplementary Figure S1A) and T47D (Figure 1C and Supplementary Figure 1B) cells resulted in reduced protein and mRNA levels of the same Notch pathway genes. These data suggest that BRCA1 may impact on Notch signalling through simultaneous transcriptional upregulation of both receptors and ligands, although transcriptional upregulation does not necessarily mean activation. Therefore, to demonstrate that BRCA1 could activate the Notch pathway, we used a β-globin promoter reporter construct (pSH2), which contains a CSL/Notch responsive element shown to be a reliable readout of NICD-mediated gene transcription (26). A JAG1 mimicking peptide (DSL) was used to exogenously activate Notch receptors. We only observed optimal DSL-mediated Notch activation in cells expressing wild-type BRCA1 [Figure 2A (i) and (ii)]. Conversely, knockdown of BRCA1 in MCF-7 and T47D cells resulted in reduced DSL-mediated activity (Figure 2B and C). Together, these data support the theory that BRCA1, through its ability to transcriptionally upregulate Notch ligands and receptors, facilitates Notch signalling and ensures that a Notch transcriptional program is functional in normal breast tissue.

### BRCA1 activates JAG1 but not Notch1 through a ΔNp63-dependent mechanism

**Figure 3.**
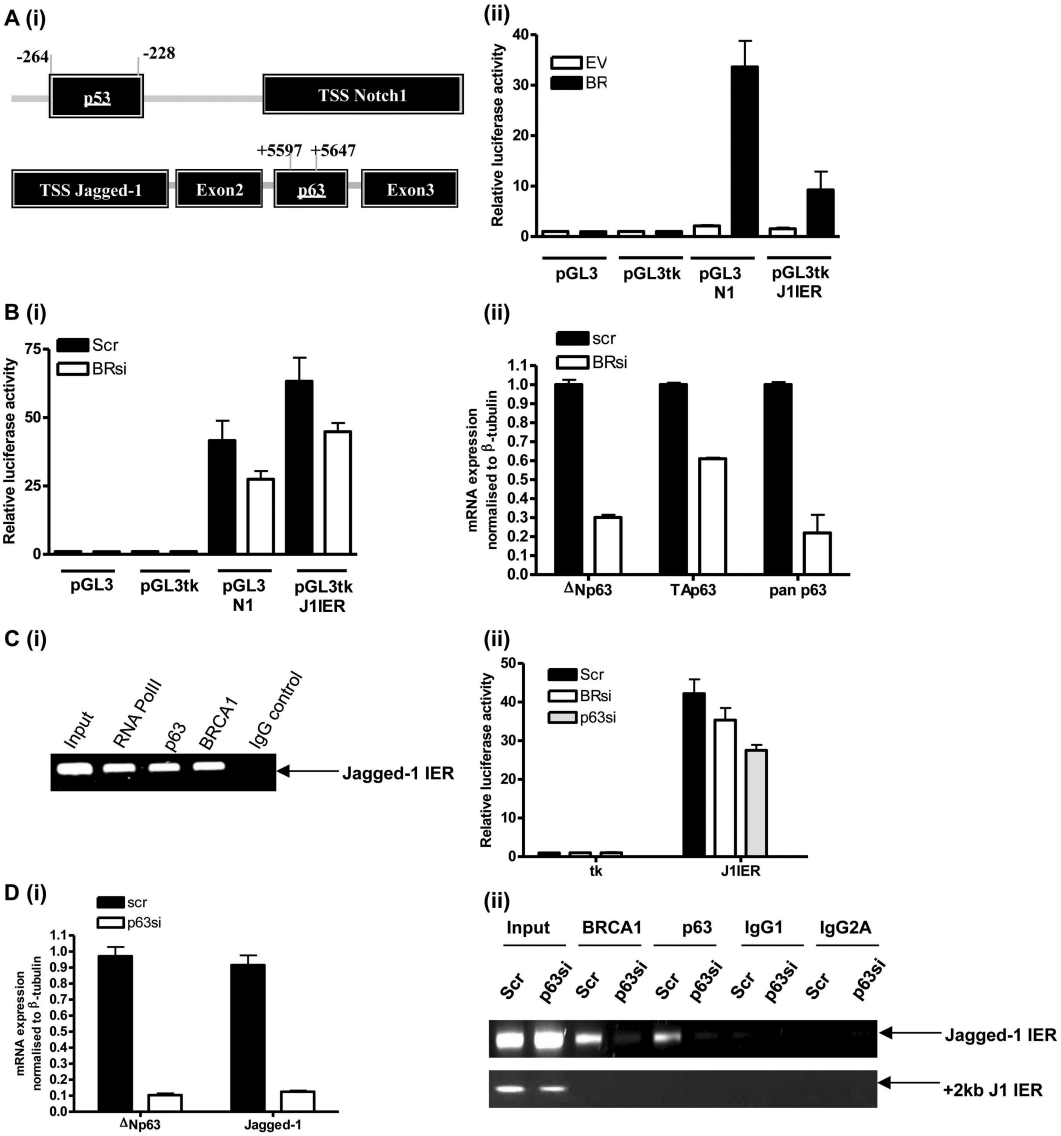
(**A**) (i) Diagram showing the p53- and p63-binding sites of the Notch1 and JAG1 genes relative to the respective transcriptional start sites (TSS). (ii) Luciferase reporter assay of HCC EV and BR cells following transfection of either a Notch1 promoter (pGL3N1) or JAG1 enhancer (pGL3tk J1IER) reporter constructs, co-transfected with a Renilla luciferase control construct. (**B**) (i) Luciferase assay of MCF-7 cells using the same reporter constructs following scrambled (Scr) control or BRCA1 (BRsi)-specific siRNA treatments. (ii) RqPCR of MCF-7 cells showing mRNA levels of pan-, TA- and ΔN- p63 transcripts following scrambled (Scr) control or BRCA1 (BRsi) specific siRNA. (**C**) (i) ChIP assay showing localization of RNA polymerase II (RNA Pol II), p63 and BRCA1 on the JAG1 enhancer (Jagged1-IER). One percent of sonicated lysate before immunoprecipitation was used as positive control (input) and IgG pulldown used as negative control (IgG control). (ii) Luciferase assay of MCF-7 cells using the JAG1 enhancer reporter (J1IER) co-transfected with a Renilla luciferase control construct following scrambled (Scr) control, BRCA1 (BRsi) or p63 (p63si) siRNA. (**D**) (i) RqPCR of MCF-7 cells showing knockdown of ΔNp63 and Jagged-1 following treatment with scrambled (Scr) control or ΔNp63 (ΔNp63si) siRNA. (ii) ChIP assay showing recruitment of BRCA1 and p63 on the JAG1 enhancer (Jagged1-IER) following scrambled (Scr) control or total p63 (p63si) siRNA. Positive and negative controls are as outlined for (C) (i). In addition, PCR using primers specific for a region upstream (+2 kb J1ER) of the JAG1 enhancer shows the specificity for the Jagged1-IER.

Next, we wanted to define the mechanism basis of Notch activation by BRCA1. BRCA1 cannot bind DNA itself and must be recruited to promoters through its ability to bind to other transcription factors. We have recently identified ΔNp63γ as a BRCA1 interacting protein, an interaction that appears to be important for the transcriptional regulation of a number of key BRCA1 target genes including p63 itself, which BRCA1 upregulates through a positive autoregulatory loop (19). Notch1 is known to be a p53 target gene (27), whereas JAG1 is specifically a transcriptional target of p63 (not p53) and contains a number of p63 responsive elements located in intron 2 (23). Notch1 and JAG1 were therefore selected for further investigation to define the mechanism underpinning their regulation by BRCA1. We generated luciferase reporter constructs containing the p53 responsive element of the Notch1 promoter (pGL3N1) and the p63 response elements in intron 2 region of the JAG1 gene cloned upstream of a minimal thymidine kinase promoter (pGL3tkJ1) [location of both regions shown in Figure 3A (i)]. In agreement with findings from Figures 1 and 2, we found that both constructs were regulated in a BRCA1-dependent manner [Figure 3A (ii) and B (i), respectively]. As HCC1937 cells possess a mutant truncated p53, we hypothesised that the BRCA1 regulation of both genes may be dependent on p63, not p53, as these often bind to the same response elements in promoters. SiRNA knockdown of BRCA1 in MCF-7 cells resulted in a significant reduction in p63 mRNA species, consistent with our previous findings [Figure 3B (ii)]. Using ChIP assay, we could demonstrate the localization of both BRCA1 and p63 to the JAG1 internal enhancer region, IER [Figure 3C (i)]. Indeed, siRNA depletion of p63 resulted in a significant reduction in JAG1-IER reporter activity, comparable with BRCA1 siRNA [Figure 3C (ii)] with equivalent reduction of JAG1 mRNA following ΔNp63 and BRCA1 siRNA but less significantly with TAp63 siRNA (Supplementary Figure S2A). We could demonstrate by ChIP assay that BRCA1 required p63 for recruitment to the JAG1-IER, as siRNA knockdown of ΔNp63 [Figure 3D (i)] resulted in loss of BRCA1 recruitment to this region [Figure 3D (ii)]. Finally, we showed that both BRCA1 and ΔNp63 are required for optimal expression of JAG1 [Supplementary Figure S2B].

Preliminary work investigating BRCA1 regulation of the Notch1 promoter has suggested an alternative mechanism may be involved, as p63 knockdown does not appear to alter Notch receptor levels and although BRCA1 is localized on the Notch promoter, p63 is not [Supplementary Figure S3A (i) and (ii)]. This reflects the fact that both p63 and JAG1 are basally restricted genes, whereas Notch receptors 1–3 are associated with the luminal layer in breast ducts [also supported by RqPCR analyses of Notch1-3 in a breast cancer cell line panel, Supplementary Figure S3B (i–iii)]. BRCA1 regulation of Notch1 is independent of p53 [Supplementary Figure S3C (i) and (ii)], and our preliminary RqPCR, luciferase and ChIP data suggest that this may be dependent on the luminal gene, GATA3 (28) [Supplementary Figure S4A (i–iii)]. This consistent with our recent findings where we have shown that BRCA1 interacts with GATA3 and represses the expression of basal genes in a GATA3-dependent fashion (29). Taken together, these experiments define the mechanistic basis of JAG1 regulation showing that BRCA1 activates JAG1 transcription through a p63-dependent process, whereas BRCA1 regulates the Notch receptors through a p63- (and p53)-independent mechanism, possibly involving GATA3.

### BRCA1 may regulate breast stem/progenitor function through Notch signalling

**Figure 4.**
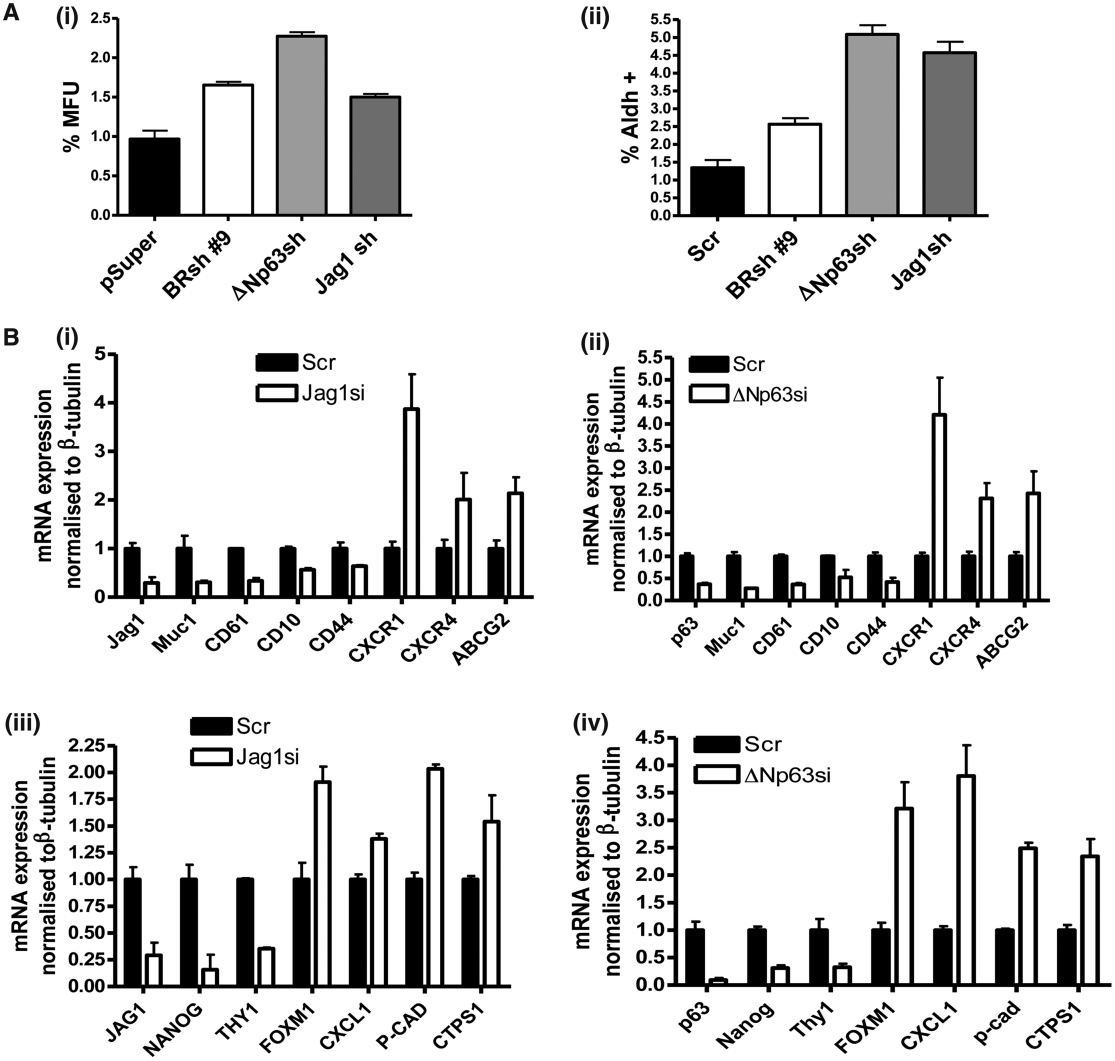
(**A**) (i) Tumoursphere cultures of MCF-7 cells following infection with retroviruses containing shRNA constructs for control (pSuper), BRCA1 (BRsh#9), ΔNp63 (ΔNp63sh) and JAG1 (JAG1sh) knockdowns. Tumourspheres were counted and expressed as %Mammary Forming Units (MFUs) relative to pSuper. (ii) The same MCF-7 shRNA cell lines were assayed for Aldefluor activity using ALDEFLUOR and expressed as percentage Aldefluor-positive (%ALDH+). (**B**) RqPCR of luminal and basal markers in MCF-7 cells treated with (i) scrambled (Scr) control or JAG1 siRNA, (ii) scrambled (Scr) control or ΔNp63 siRNA. RqPCR of stem cell and basal-like markers in MCF-7 cells treated with (iii) scrambled (Scr) control or JAG1 siRNA or (iv) scrambled (Scr) control or ΔNp63 siRNA. β-tubulin mRNA was used for normalization.

**Figure 5.**
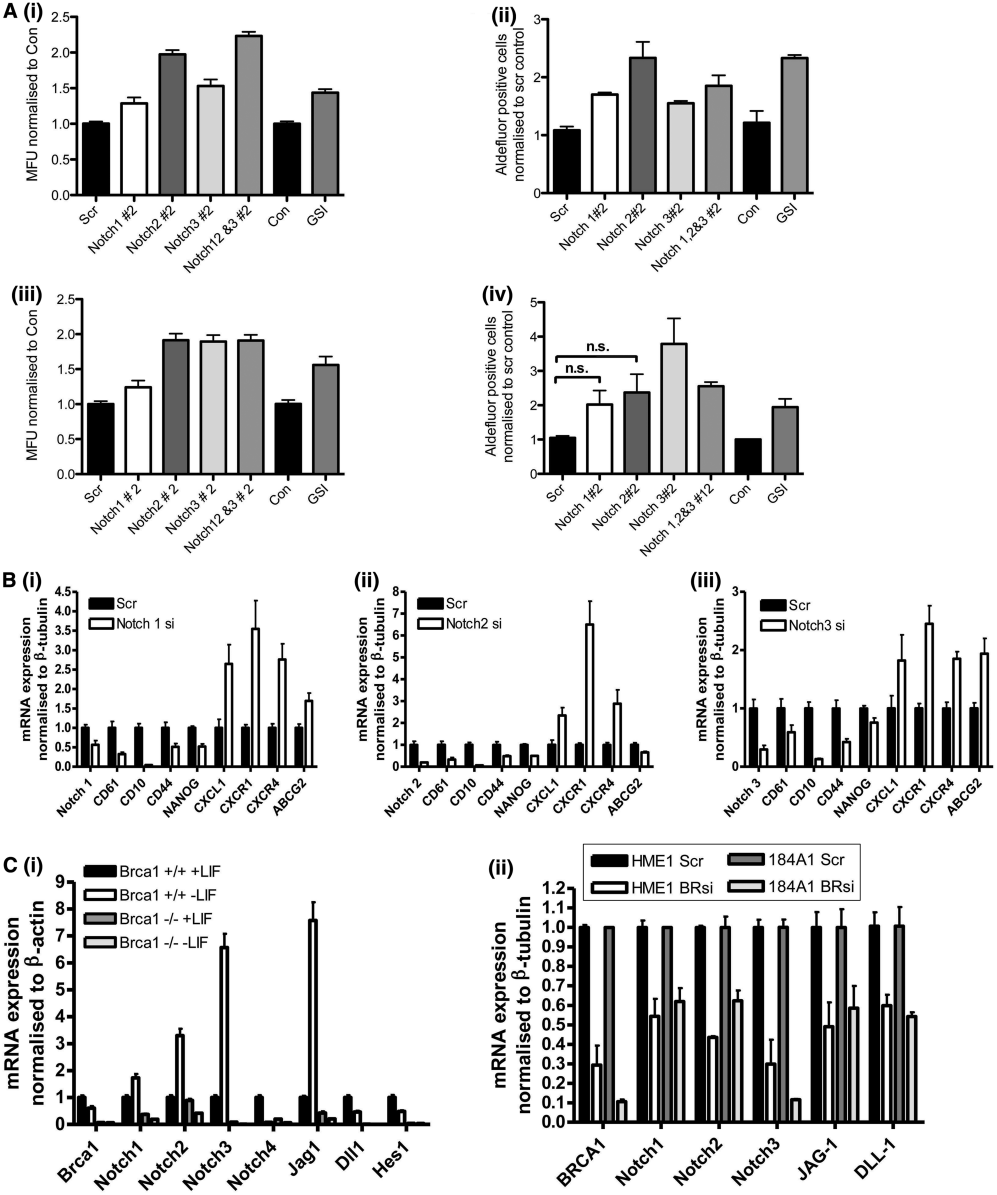
(**A**) Tumoursphere cultures of (i) MCF-7 or (iii) T47D cells treated with Scrambled (Scr), Notch1 (Notch1#2), Notch2 (Notch2#2), Notch3 (Notch3#2), a combination of Notch1, 2 and 3 (Notch1,2&3#2) or Gamma Secretase Inhibitor (GSI). Tumourspheres were counted and expressed as % Mammary Forming Units (MFUs) relative to control. Aldefluor activity assay of the same (ii) MCF7 and (iv) T47D cells with % Aldefluor positive cells calculated and shown. (**B**) RqPCR of lineage and stem cell markers in MCF-7 cells treated with Scrambled (Scr) or (i) Notch1 (Notch1 si), (ii) Notch2 (Notch2 si) or (iii) Notch3 (Notch3 si) siRNA. (**C**) (i) RqPCR for components of the Notch pathway in Brca1 wild-type (Brca1 +/+) and Brca1 null (Brca1 −/−) murine ES cells grown in the presence (+LIF) or absence (−LIF) of LIF for 4 days. (ii) RqPCR for components of the Notch pathway in HME1 and 184A1 normal breast cell lines transiently transfected with either scrambled (Scr) or BRCA1 (BRsi)-specific siRNA.

BRCA1 has been postulated to be a regulator of breast stem cell function (6), and p63 has been implicated in the stem cell regulation of a number of different tissue types (30). We have previously shown that loss of BRCA1 and p63 in MCF-10A cells leads to an increase in stem/progenitor cells (19). We therefore wanted to investigate whether the Notch pathway acts an effector pathway downstream of BRCA1 and p63 in stem cell regulation. Knockdown of BRCA1, ΔNp63 or JAG1 in MCF-7 cells decreased Notch activity, and all resulted in enhanced tumoursphere growth (Figure 4A (i) and Supplementary Figure S5A). Indeed, monolayer cultures of MCF-7 cells with stable shRNA knockdown of these three genes all showed enhanced Aldefluor activity [Figure 4A (ii)]. Although neither tumoursphere culturing nor Aldefluor activity are wholly definitive of stem cell function, this suggests that normal differentiation process has been altered. Using RqPCR, we observed that knockdown of p63 or JAG1 correlated with loss of a number of markers associated with luminal differentiation such as muc1 and CD61 as well as basal markers such as CD10 and CD44 [Figure 4B (i) and (ii), Supplementary Figure S5B (i) and (ii)]. Knockdown of JAG1 or ΔNp63 resulted in the upregulation of proliferation-associated markers (FoxM1 and CXCL1) and markers commonly associated with basal-like breast cancer (p-cadherin, CXCL1 and CTPS1) [Figure 4B (iii) and (iv), Supplementary Figure S5B (iii) and (iv)]. This did not appear to be associated with a generalized non-specific expansion of increased stem cell markers, as expression of genes such as Nanog were actually reduced, whereas other stem cell markers like CXCR4 were increased (Figure 4B and Supplementary Figure S5B). Using the cell surface markers CD49f, EpCAM and CD24 (21), we observed a decrease in progenitor and mature populations with an increase in the putative stem cell population (Supplementary Figure S6A). We did note, however, the intensity of the CD49f staining was decreased compared with scrambled control, indicating that this may represent an aberrant expanded population distinct from the classical CD49f high stem cell population. Inhibition of the Notch pathway in MCF-7 and T47D cells through either Notch receptor siRNA or the use of chemical inhibitors (GSI) showed similar effects on tumoursphere formation, Aldefluor activity and changes in marker expression observed following JAG1 and ΔNp63 siRNA, suggesting that Notch signalling is important for luminal-basal cell fate determination in breast tissue (Figure 5 and Supplementary Figure S6).

To investigate whether this mechanism was a general phenomenon and also present in non-cancerous normal cells, we used murine ES cells lacking wild-type Brca1 (20) grown in the presence and absence of leukemia inhibitory factor (LIF) (to prevent and promote differentiation, respectively). We observed that Brca1 regulated the expression of both basal and luminal components of the Notch signalling pathway in normal cells [Figure 5C (i)]. In contrast to the more differentiated breast cancer cell lines, Notch4 was readily detectable in the ES cells, whereas Notch3 expression levels were difficult to detect, reflecting the known expression patterns and functional roles of these two genes in differentiation and development (14). To look more specifically at BRCA1-dependent regulation of the Notch receptors and ligands in the normal breast, we used two pseudo-normal breast cancer cell lines—HME-1 h-TERT immortalized cells and spontaneously immortalized 184A1 cells. siRNA-mediated knockdown of BRCA1 resulted in downregulated expression of Notch receptors and ligands [Figure 5C (ii)] consistent with our results from breast cancer cell lines and the ES cells. Next, we wanted to extend our studies into patient samples, as siRNA knockdown of genes in cell lines may not adequately represent the complex and heterogeneous nature of BRCA1 mutations *in vivo* including interactions of BRCA1 mutant breast cells with stromal and immune compartments. We therefore used gene expression data from a small in-house data set of 10 BRCA1 mutant and 10 sporadic breast cancer patients as well as a publically available data set containing BRCA1 mutant and sporadic breast cancer cases (31). Consistent with our results from cell line models, we observed decreased expression of Notch pathway signalling components and differentiation markers in BRCA1 mutant profiles, whereas the expression of stem cell-like, proliferation and basal-like markers were increased (Supplementary Figure S7). Together, we conclude that Notch signalling downstream of BRCA1 is important in the ordered differentiation and growth control of basal and luminal precursors in both normal and cancerous breast tissue.

**Figure 6.**
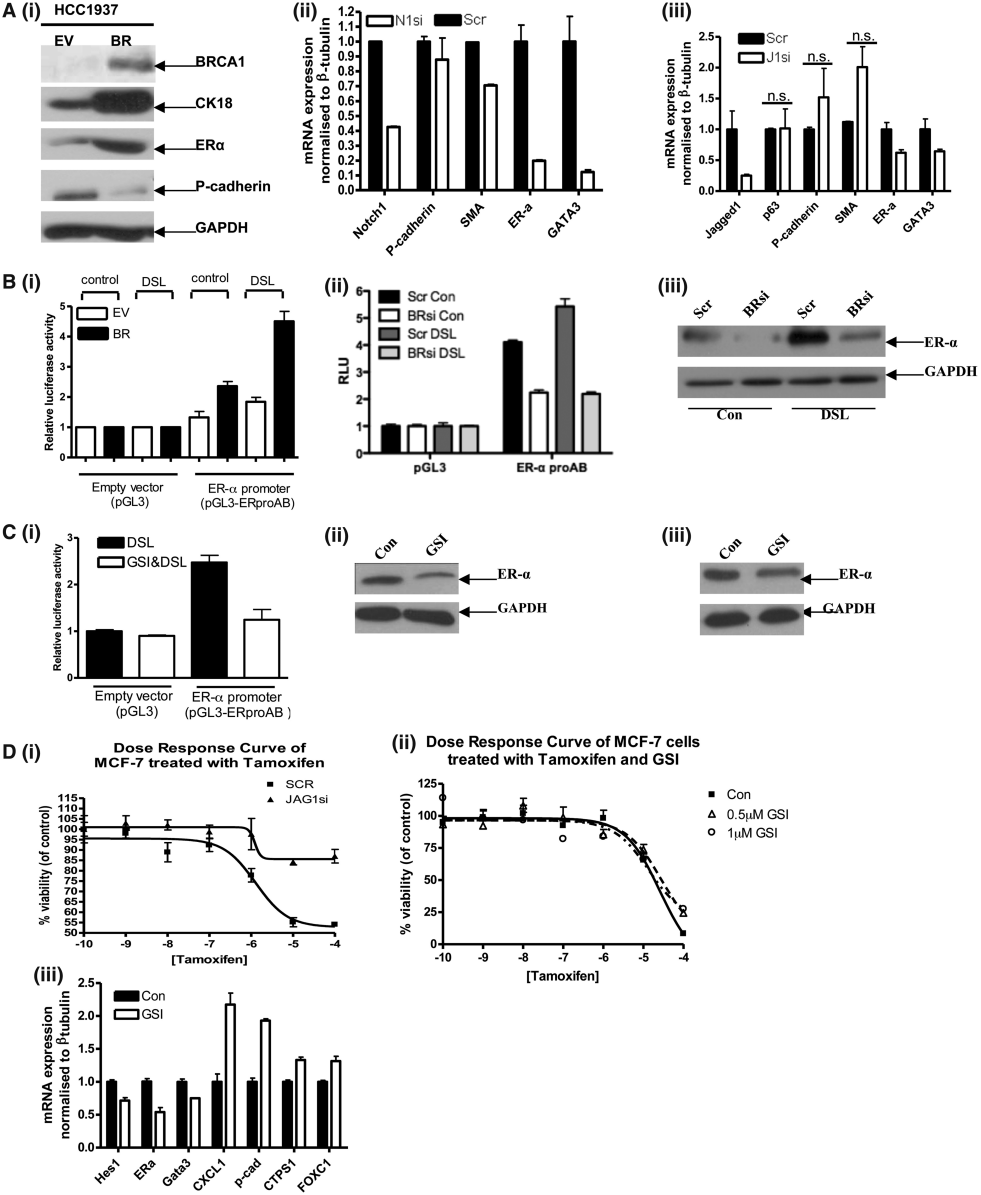
(**A**) (i) Immunoblots of HCC EV and BR cells probed with the antibodies shown and reprobed with β-tubulin as a loading control. (ii) RqPCR of MCF-7 cells showing mRNA levels of basal and luminal markers following scrambled (Scr) control or Notch1 (N1si)-specific siRNA. (iii) RqPCR of MCF-7 cells showing mRNA levels of the same basal and luminal markers following scrambled (Scr) control or JAG1 (J1si)-specific siRNA. (**B**) (i) Luciferase reporter assay of HCC EV and BR cells following transfection of either an empty vector (pGL3) or a reporter vector containing the AB regions of the ERα promoter (pGL3-ERproAB), co-transfected with a Renilla luciferase control construct. Cells were treated with either a scrambled peptide (control) or a Notch ligand mimicking peptide (DSL) before assay. (ii) Luciferase reporter assay of T47D cells using the same reporter contructs pre-treated with either scrambled control (Scr) or BRCA1 (BRsi)-specific siRNA Cells were treated with either a scrambled peptide (Con) or a Notch ligand-mimicking peptide (DSL) before assay. (iii) Western blot of MCF7 cells treated with scrambled (Scr) or BRCA1 (BRsi)-specific siRNA followed by either a scrambled peptide (Con) or Notch ligand-mimicking peptide (DSL). Blots were then probed for ER-α and GAPDH as a loading control. (**C**) (i) Luciferase reporter assay of MCF-7 cells using the same reporter constructs as (B), but instead cells were treated with either DSL alone or DSL combined with a Gamma Secretase Inhibitor (GSI&DSL). Immunoblot of (ii) MCF-7 or (iii) T47D cells treated with Gamma Secretase Inhibitor (GSI) or vehicle control (Con) for 24 h. Blots were probed with ER-α or GAPDH as a loading control. (**D**) (i) Dose response curve of MCF-7 cells treated with different doses of Tamoxifen (Log_10_ M) (for 72 h) following treatment with scrambled (SCR) control or JAG1 (JAG1si)-specific siRNA. (ii) Dose response curve of MCF-7 cells treated with different doses of Tamoxifen (Log_10_ M) following pre-treatment with Gamma Secretase Inhibitor (GSI). (iii) RqPCR of MCF-7 cells treated with either Gamma Secretase Inhibitor (GSI) or vehicle control (Con) for 24 h.

Our colleagues have previously shown that BRCA1 transcriptionally regulates a number of basal and luminal terminal differentiation markers at the promoter level, determining commitment to a particular cell fate within the mammary gland (32). For example, exogenous expression of functional BRCA1 in BRCA1 mutant cells results in the reduced expression of basal genes, such as p-cadherin and the upregulation of luminal genes such as cytokeratin 18 and ER-α [Figure 6A (i)]. Notch signalling has also been implicated in mammary epithelial cell differentiation in the murine mammary gland, and the Notch target gene GATA3 is strongly associated with the expression of the luminal marker ER-α in mammary epithelial cells and for maintaining luminal characteristics in breast cells (33). We wanted to assess the functional consequences of Notch1 and JAG1 downstream of BRCA1 in mammary epithelial cell fate. Using knockdown of both genes in MCF-7 cells, we found that both were required for the expression of luminal genes GATA3 and ER-α. [Figure 6A (ii) and (iii)]. Notch1 expression was not required for suppression of the basal genes smooth muscle actin and p-cadherin, whereas JAG1 knockdown was accompanied by upregulation of both these basal marker genes (in agreement with the restricted expression of JAG1 to basal epithelia), though this was not statistically significant. To test the link between Notch activation and the expression of luminal marker genes, we used a reporter construct containing the proximal AB promoter regions (proAB) of the ER-α gene (7). BRCA1 mutant cells were unable to drive transcription from this promoter even in the presence of DSL treatment, whereas cells expressing wild-type BRCA1 showed a significant increase in activation over treatment with a control peptide [Figure 6B (i)]. Similar results were also seen using siRNA knockdown of BRCA1 in MCF-7 and T47D cells [Figure 6B (ii) and (iii)]. Alternatively, as Figure 6C (i) shows, activation of the ER-α promoter construct in MCF-7 cells with DSL stimulation was almost completely abolished by treatment with the γ-secretase inhibitor. Similar results were also seen at a protein level in MCF-7 and T47D cells (Figure 6C (ii) and (iii), respectively). Having shown the importance of Notch signalling in luminal differentiation and ER-α expression, we wanted to investigate whether the therapeutic use of Notch pathway inhibitors, such as γ-secretase inhibitors, would alter the response to anti-estrogen therapy. Inhibition of the Notch pathway by siRNA-mediated knockdown of JAG1 (Supplementary Figure S8A) in MCF-7 cells resulted in an impaired growth inhibitory response to the antiestrogen, Tamoxifen [Figure 6D (i)], suggesting a loss of ER-α dependent signalling. The use of a γ-secretase inhibitor in combination with tamoxifen did not appear to produce even additive effects and actually appeared to slightly (but significantly) protect MCF-7 cells at higher tamoxifen concentrations [Figure 6D (ii)]. Similar results were also observed using T47D cells (Supplementary Figure S8B–D). In addition, the use of a γ-secretase inhibitor led to the loss of ER-α and GATA-3 expression as well as an observed increase in the expression of proliferation markers (CXCL1) and basal-like breast cancer markers (p-cadherin, CTPS1 and FOXC1) [Figure 6D (iii)]. Together, these data highlight (i) the importance of Notch signalling downstream of BRCA1 and suggest an interdependence between Notch and estrogen signalling to maintain the luminal phenotype and (ii) how inhibition of Notch signalling may lead the disruption of normal mammary differentiation leading to the emergence of more aggressive basal-like cancer subtypes.

## DISCUSSION

In this study, we show that BRCA1 activates the Notch pathway in breast cells by transcriptionally upregulating Notch ligands and receptors in both normal and cancer cells. We show the mechanism through which BRCA1 regulates the Notch ligand JAG1, an event that is dependent on ΔNp63 expression. Knockdown of either Notch1 or JAG1 phenocopies BRCA1 knockdown resulting in the loss of ERα and luminal marker expression. We show that we can drive ERα expression through Notch activation, whereas a γ-secretase inhibitor reversed this process and abrogated the ability of cells to respond to ERα targeted therapy. Knockdown of BRCA1, ΔNp63 or JAG1 in MCF-7 cells resulted in increased tumoursphere growth and ALDH1 activity. Knockdown of Notch signalling components in luminal cells also resulted in decreased expression of some stem and progenitor markers but upregulation of proliferation-associated markers and markers of basal-like breast cancer. This is accompanied by an increase in a stem cell-like population as shown by flow cytometry. Together, these findings suggest that BRCA1 regulation of Notch signalling is important in the normal differentiation process in breast tissue, and that loss of this pathway may be a key event in the progression of basal-like breast cancers.

Here, we show that Notch signalling downstream of BRCA1 may be central to normal breast differentiation, as we see regulation of the Notch pathway by BRCA1 in non-tumorigenic mammary cell lines derived from reduction mammoplasty as well as in murine ES cells. This is in keeping with the observation that the Notch signalling pathway was differentially expressed in cells from BRCA1 mutation carriers when compared with wild-type (9). It is also evident from a number of other studies that Notch signalling must also be tightly controlled. Overexpression of NICD1 or RBP-Jκ results in the transformation of normal breast epithelial cells (34), whereas Notch3 has been shown to play an important tumour suppressive role through its ability to upregulate p21^cip1/WAF1^ and induce senescence (35). Activation of Notch signalling in ERα-negative breast cells has been linked with the suppression of apoptosis through upregulation of survivin (36), inferring that the increased sensitivity of ERα-negative breast cells to Notch inhibition was possibly due to their ‘addiction’ to the Notch pathway (36). Simultaneous inhibition of EGFR and Notch activity has been shown to result in a synthetic lethality in basal-like breast cancer (37). Rizzo and colleagues also showed that ERα signalling actually inhibited Notch receptor activity and Notch NICD nuclear levels, an effect reversed following treatment with tamoxifen (38). In our study, however, we observe low/absent Notch signalling in ERα-negative breast cells as well as in BRCA1 mutant cell lines. Our study contradicts the findings of the studies described earlier in the text for a number of reasons including (i) the fact that these studies used overexpressed NICD1 (which does not reflect the normal physiological scenario); (ii) instead of targeting specific Notch receptors, such studies performed pan-Notch inhibition with γ-secretase inhibitors (which will also inhibit TGFα cleavage and hence EGFR activation) (36); and (iii) the studies of Haughian *et al.* (39) and Rizzo *et al.* (40) use models of anti-estrogen therapy resistance, whereas we use therapy naïve cells. In addition, ERα-negative breast cancers are known to show a high degree of heterogeneity and have been subclassified into at least two different subgroups (41). It is likely that not all ERα-negative breast cancers share dysfunctions in BRCA1 signalling and that intrinsic subgroups may show normal or indeed constitutively active Notch signalling. Furthermore, the Notch pathway has been shown to act downstream of p53 in the repression of mammary stem/progenitor cells (42). Therefore, the p53 mutational status of the cell lines models used in studies may also partly explain discrepancies between our results and others.

JAG1 expression has been shown to be restricted to basal cells and its overexpression has been linked to basal-like breast cancer (43). BRCA1 mutant breast tumours have been reported to express higher levels of JAG1 mRNA compared with BRCA2 mutant tumours; however, this was not significant (44). Contrary to these observations, we consistently observed a positive association between BRCA1 function and JAG1 expression in both cell lines and patient samples. Although our BRCA1 knockdown and reconstitution studies in cell lines are consistent, reliance on *in vitro* models may not accurately reflect the *in vivo* scenario where cell–stromal interactions and immunoediting will significantly impact on tumour behaviour. Using patient data, we observed that the correlation between BRCA1 mutational status and expression of Notch receptors and ligands (in addition to some of the downstream differentiation and proliferation genes highlighted) is stronger in our in-house data set compared with the Pawitan data set (31). This may reflect the fact that in our in-house data set, we were able to stratify BRCA1 mutant profiles based on triple negativity, and these tumours were therefore more likely to consistently display the ‘BRCAness’ phenotype (45). It must be considered that although triple negativity is enriched in BRCA1 mutant profiles, not all BRCA1 tumours may possess a triple negative phenotype. In the Pawitan data set, this information was not available, and therefore our comparison may have included a small number of BRCA1 mutant tumours expressing one or several of ERα, PR or HER2, in addition to sporadic cases possessing ‘BRCAness’ characteristics, both of which will complicate any comparison.

Our data indicate that loss of the BRCA1/p63/Notch signalling axis results in an increase in an aberrant population with cancer stem cell properties. However, the decrease in some of the cancer stem cell markers such as Nanog implies that this may represent an as yet unclassified cancer stem cell population. Numerous studies have demonstrated that the cancer stem cell phenotype is heterogeneous (46) with one study showing at least two distinct populations with cancer stem cell characteristics and Notch1 expression in cell lines derived from a Brca1 knockout mouse model (47). In addition, a recent publication has demonstrated a Notch-independent population of breast cancer stem cells within the MCF-7 cell line (48) further highlighting the heterogeneity of stem cells.

Some of our findings are not in keeping with the results of Harrison *et al.* (16) who showed that genetic or pharmacologic inhibition of Notch1 (and Notch4) reduced stem cell activity. This may result from the use of different cell surface antigens to identify the stem cell-like population (EpCAM/CD44/CD24 versus EpCAM/CD49f/CD24). In agreement with our findings, Keller *et al.* (21) have shown that use of CD44 does not accurately stratify cell lines based on tumour subtype, whereas CD49f mimics the findings seen by gene expression analysis and also in primary breast samples. We observe a reduction in CD44 expression following loss of Notch signalling, suggesting that our aberrant population would not be detected by the methods used in the Harrison study. In summary, we believe the apparent BRCA1-Notch discrepancy with our study reflects the (i) heterogeneous nature of the cancer stem cell phenotype; (ii) experimental differences such as use of different techniques and cell surface antigens to identify stem-like cells; and (iii) differences in the p53 status, which can have influence on stem cell function (as discussed earlier in the text).

We also observe similar BRCA1-dependent effects following ΔNp63 knockdown. We believe this is significant finding, as our group has also recently identified the ΔNp63 family of proteins as downstream transcriptional targets of BRCA1 (19). We have demonstrated that BRCA1 interacts with ΔNp63γ and requires ΔNp63 expression to transactivate JAG1. It is known that Notch signalling and ΔNp63 expression are mutually antagonistic, and the two pathways act to control the luminal and basal compartments, respectively, in breast tissue (49). We believe that the BRCA1-ΔNp63γ complex is important for transcriptionally upregulating genes involved in normal basal differentiation and for transmitting a Notch-responsive signal to adjacent cells through transcriptional upregulation of JAG1 (and probably other Notch ligands). Adjacent cells would thus adopt a Notch-responsive transcriptional program leading to the enforcement of luminal differentiation through ERα and GATA3 signalling (50). This event would appear to be important for maintaining growth control of uncommitted or bipotent progenitor cells, as loss of BRCA1, ΔNp63 or JAG1 all show similar phenotypes including loss of differentiation markers, enhanced proliferation of tumourspheres and upregulation of proliferation-associated markers. In summary, we have identified a number of Notch proteins as key transcriptional targets of the breast/ovarian cancer tumour suppressor gene BRCA1. We show that control of Notch signalling is an important aspect of BRCA1 tumour suppression with roles in the differentiation, basal-luminal lineage specification and growth control of breast tissue. These findings are likely to have implications for the management of breast cancer patients and a greater understanding of the intricacies of Notch signalling and breast development may be of great benefit in the future design of breast cancer chemotherapies.

## SUPPLEMENTARY DATA

Supplementary Data are available at NAR Online.

Supplementary Data
