# Supplementary material for: BRCA1 is a key regulator of breast differentiation through activation of Notch signalling with implications for anti-endocrine treatment of breast cancers
Source: Nucleic Acids Res. 2013 Jul 17;41(18):8601–14. doi: 10.1093/nar/gkt626 (PMC3794588; doi:10.1093/nar/gkt626)
Supplement: Supplementary Data [file supp_41_18_8601__index.html]

BRCA1 is a key regulator of breast differentiation through activation of Notch signalling with implications for anti-endocrine treatment of breast cancers — BRCA1 is a key regulator of breast differentiation through activation of Notch signalling with implications for anti-endocrine treatment of breast cancers — Supplementary Data 

# BRCA1 is a key regulator of breast differentiation through activation of Notch signalling with implications for anti-endocrine treatment of breast cancers

## 

files

**Files in this Data Supplement:**

- Supplementary Data - pdf file
