# Supplementary material for: BRCA1 is a key regulator of breast differentiation through activation of Notch signalling with implications for anti-endocrine treatment of breast cancers
Source: Nucleic Acids Res. 2013 Jul 17;41(18):8601–14. doi: 10.1093/nar/gkt626 (PMC3794588; doi:10.1093/nar/gkt626)
Supplement: Supplementary Data [file supp_gkt626_nar-02504-x-2012-File008.pdf]

Supplementary Figure 1

A

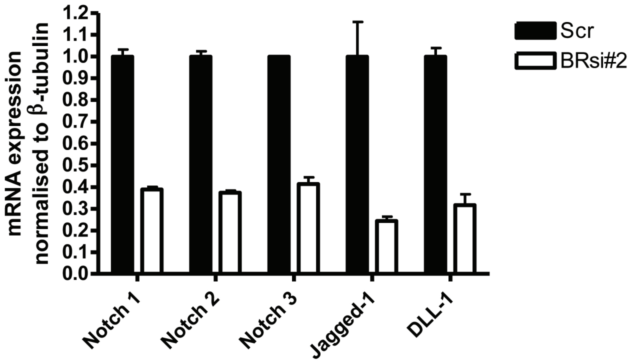

B

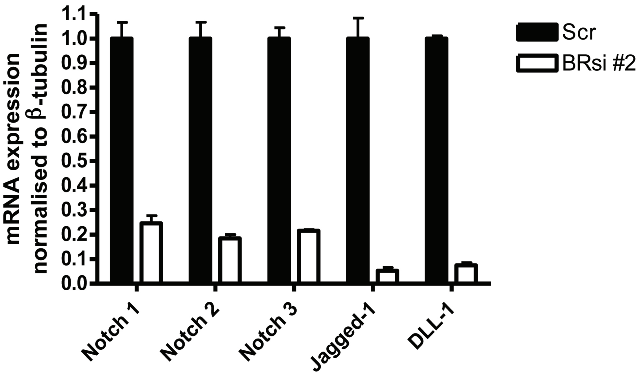

Supplementary Figure 2

A (i)

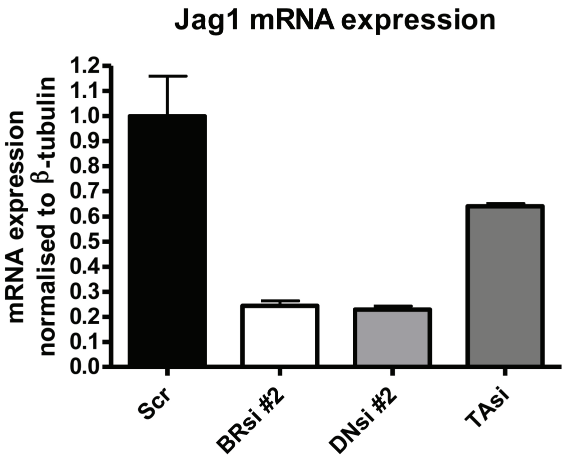

(ii)

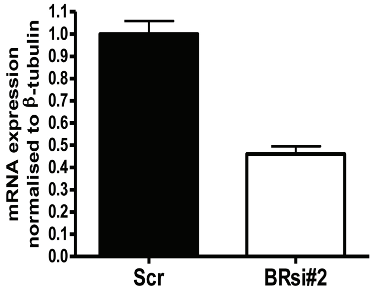

(iii)

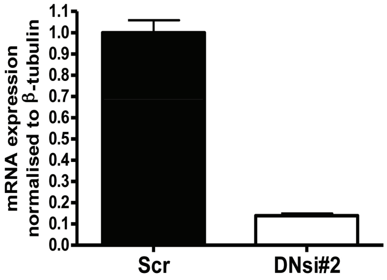

(iv)

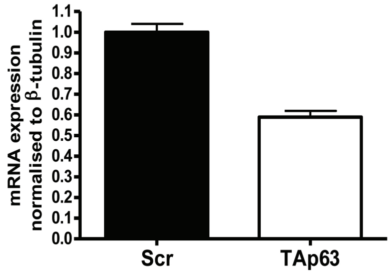

(B)

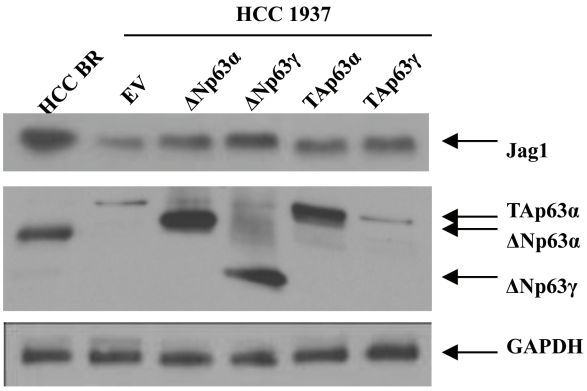

Supplementary Figure 3

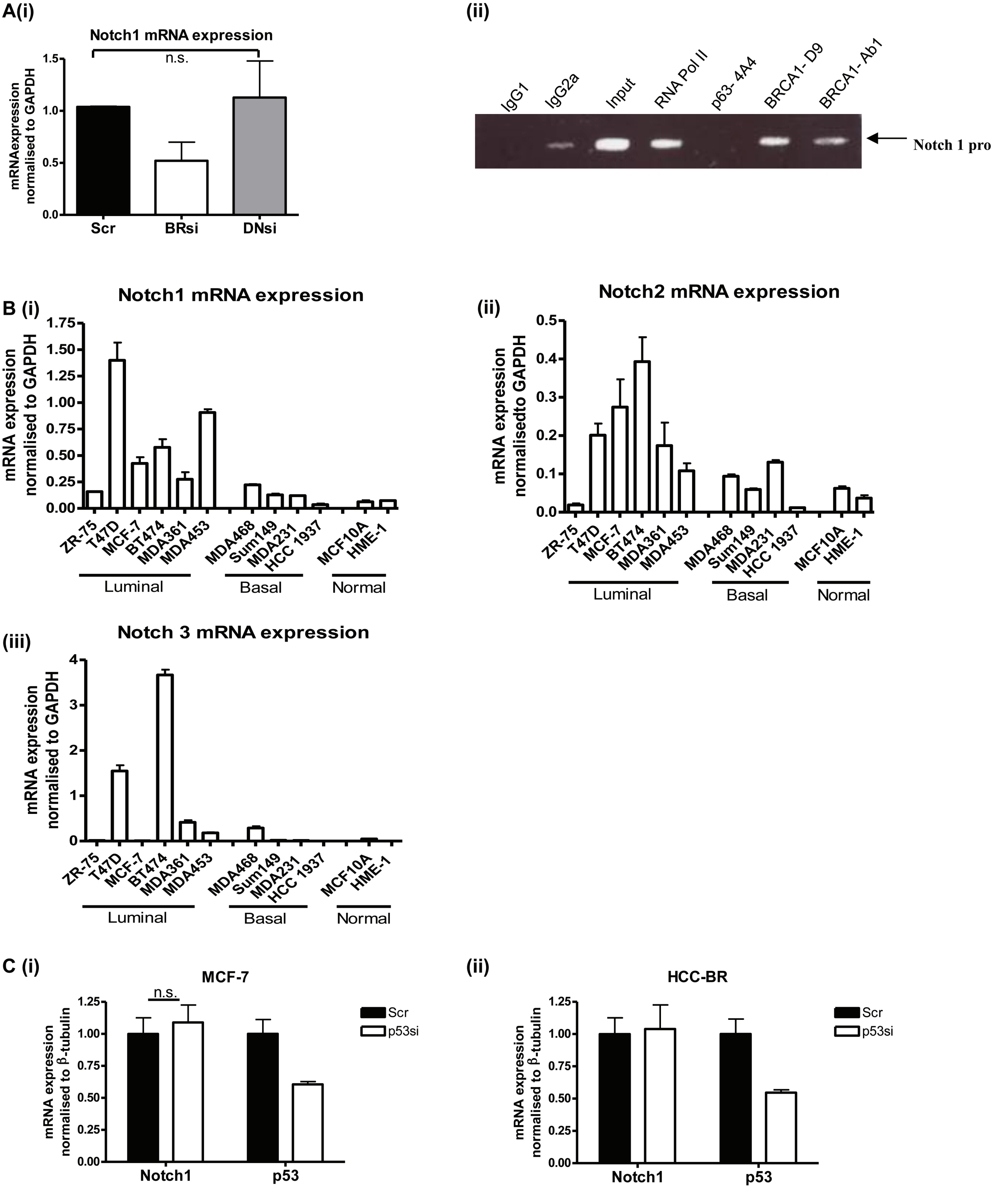

Supplementary Figure 4

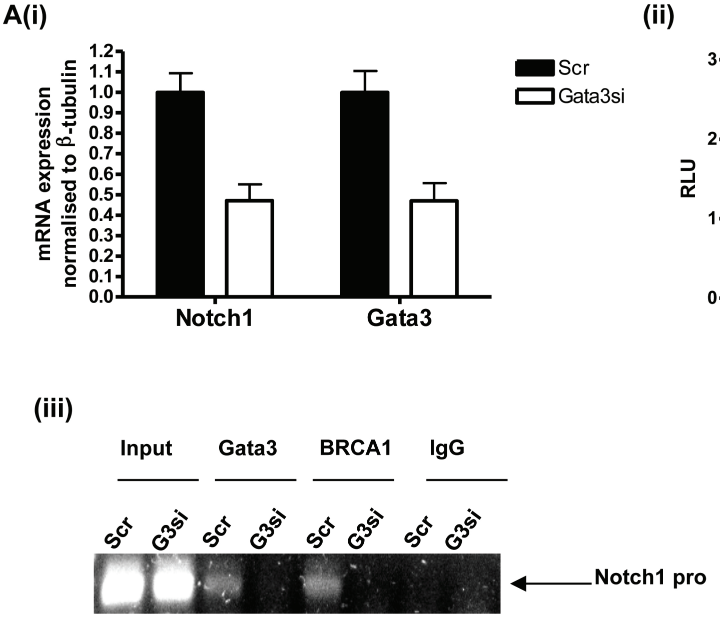

Supplementary Figure 5

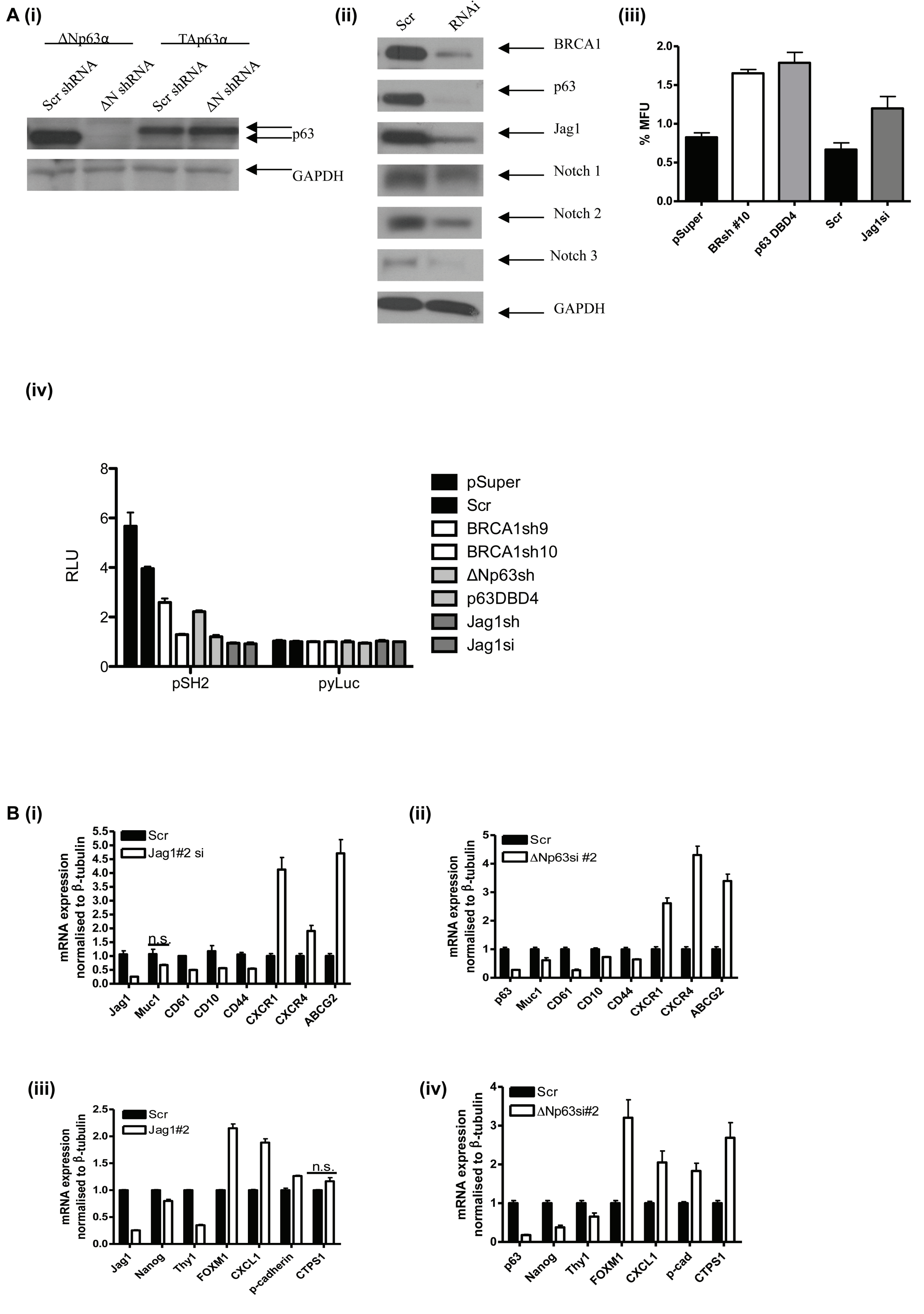

Supplementary Figure 6

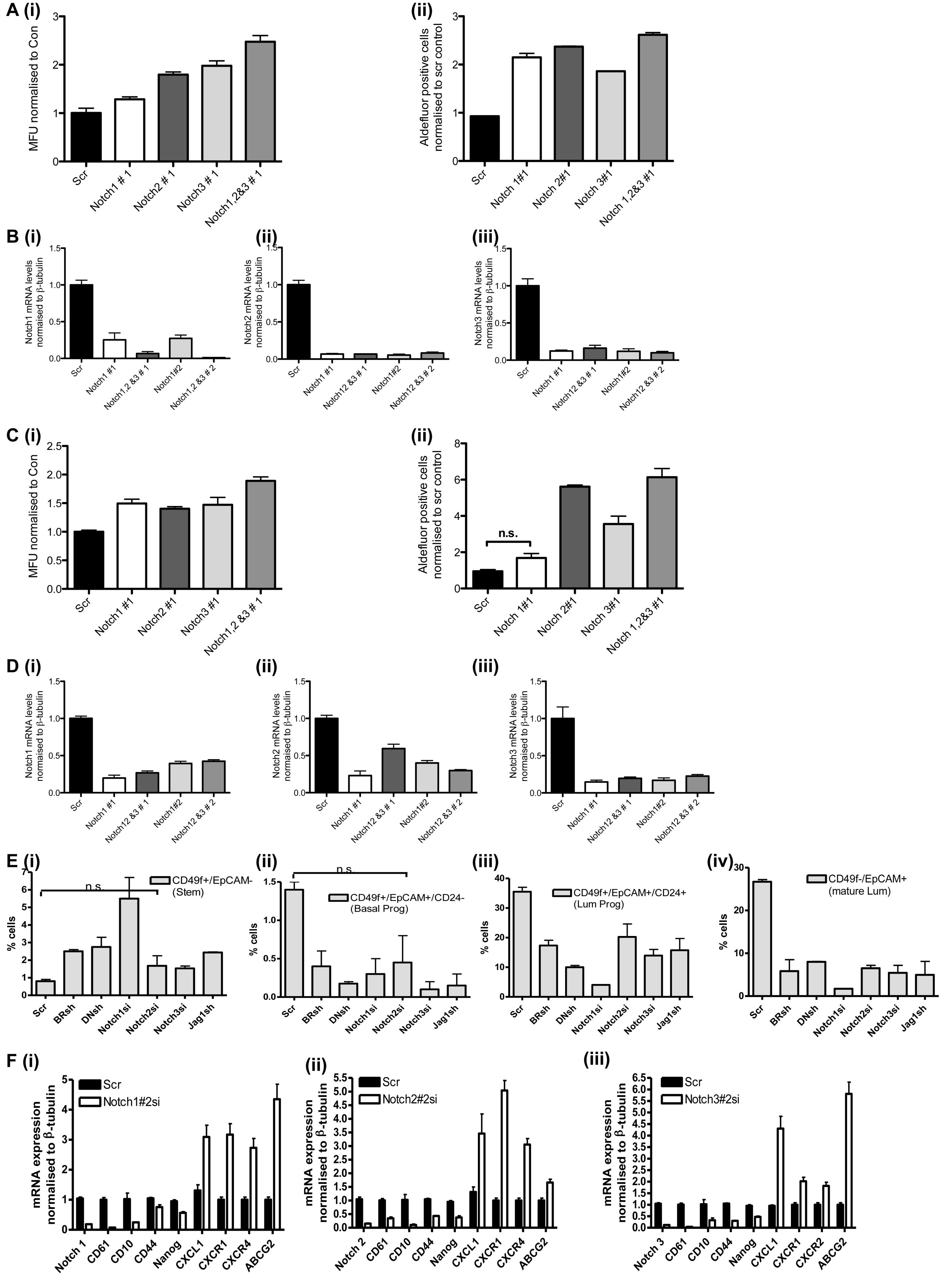

Supplementary Figure 7

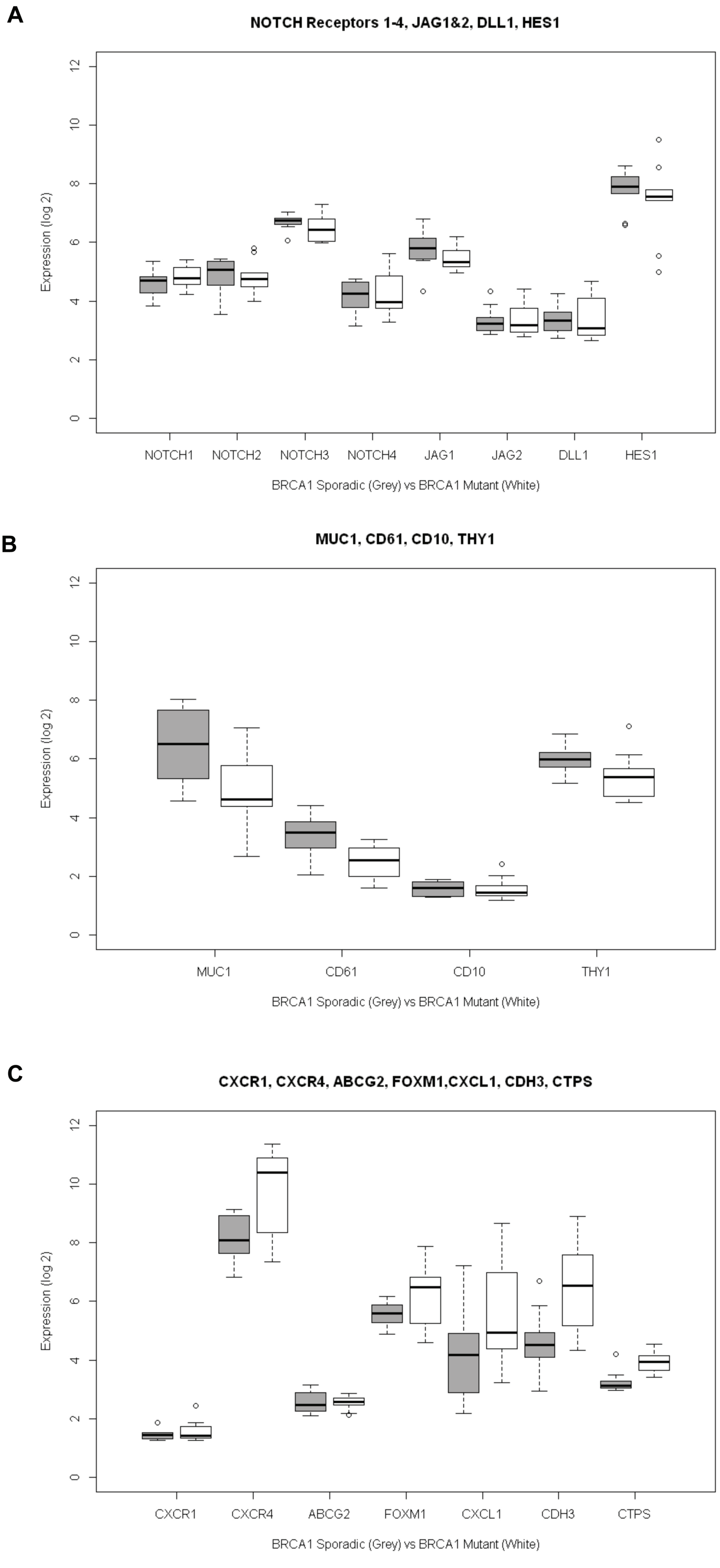

Supplementary Figure 7 contd

D

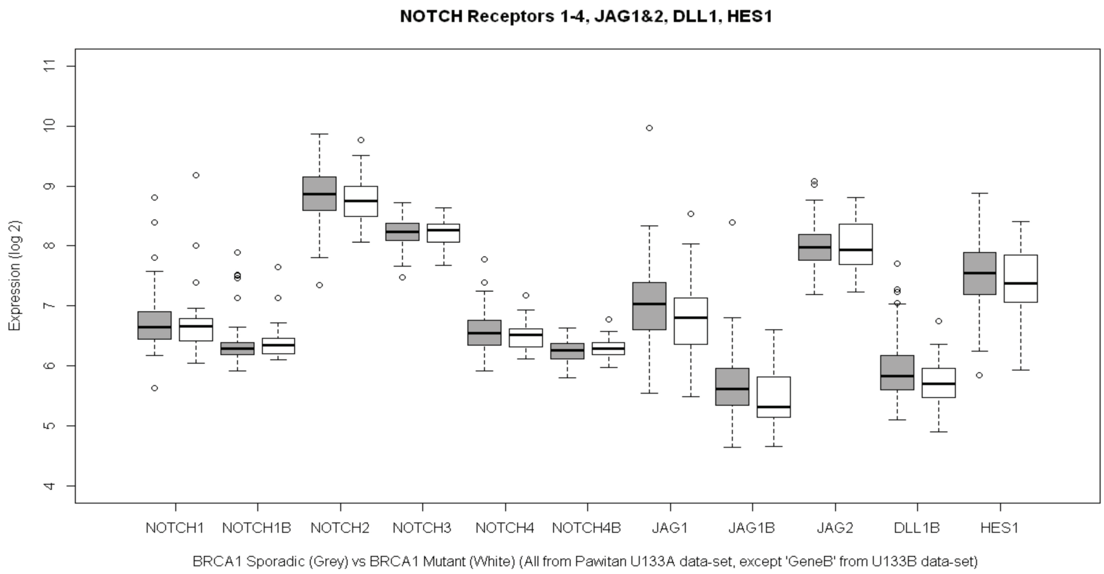

E

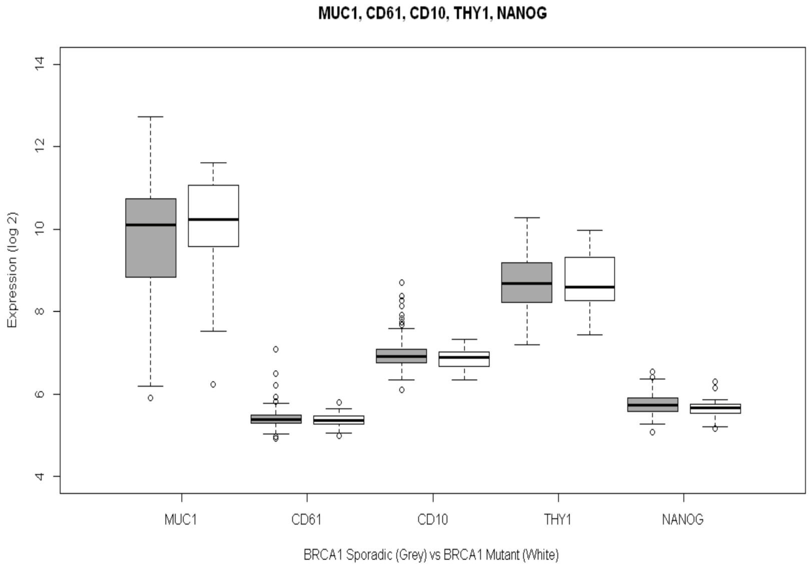

F

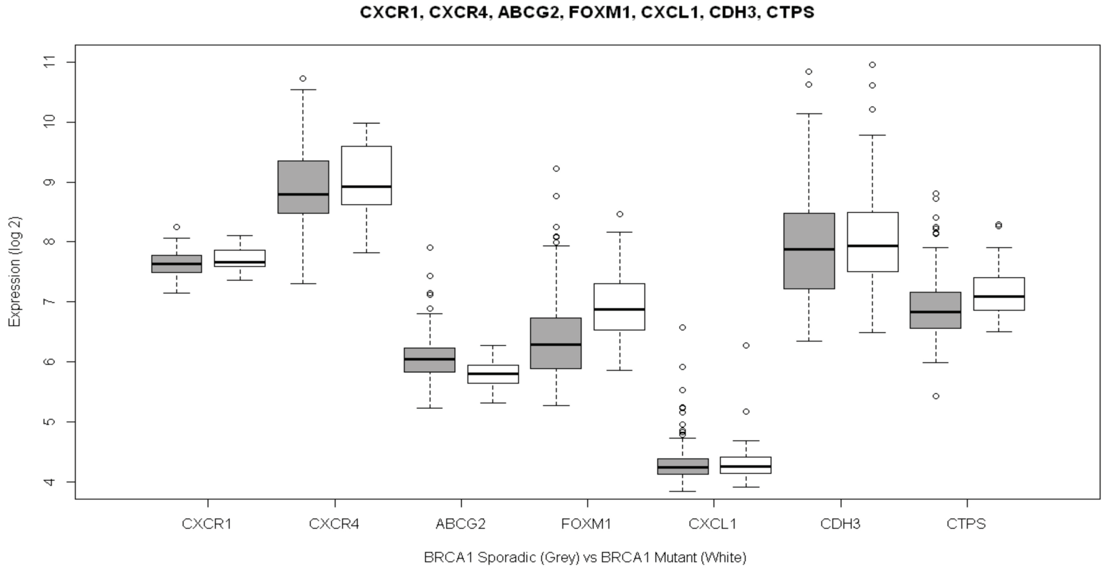

Supplementary Figure 8

A

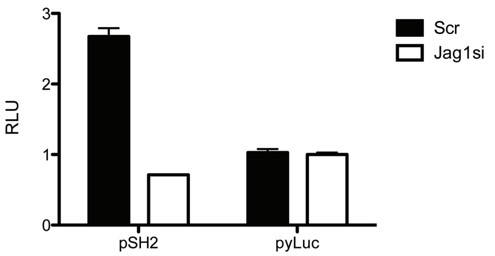

B

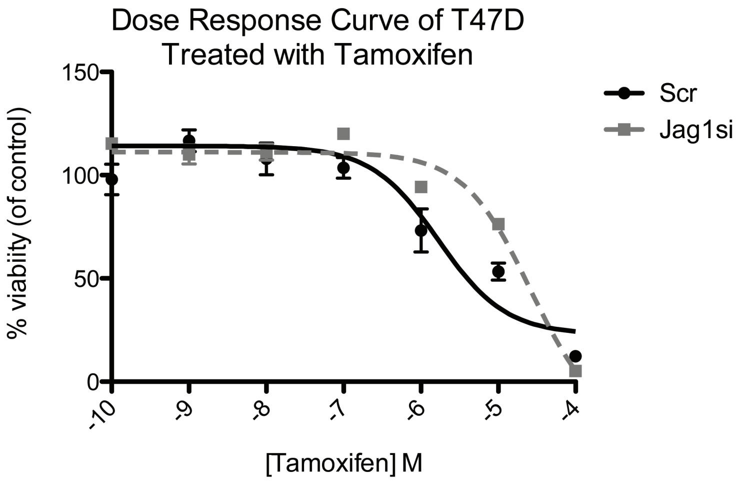

C

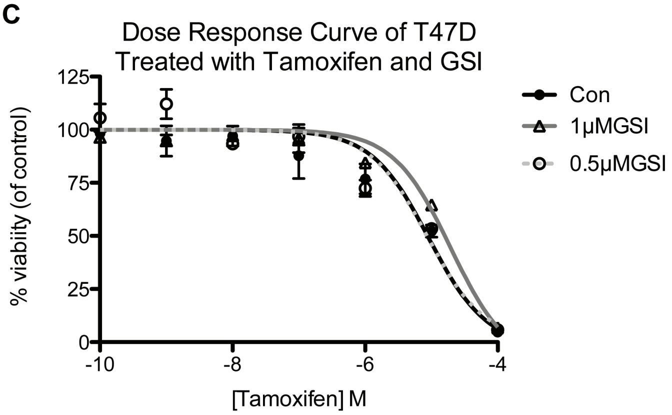

D (I)

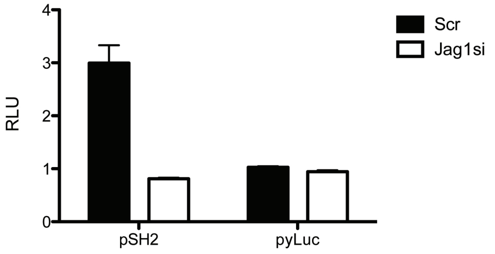

(ii)

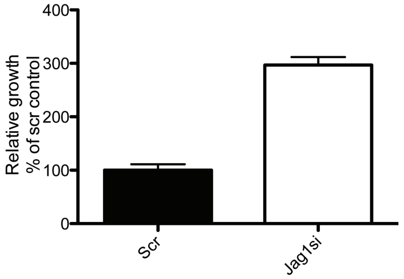

Supplementary Figure Legends
Dataset S1. Table of p values for statistical analysis throughout the manuscript.

Supplementary Figure 1. Real time PCR analysis of Notch receptors and ligand mRNAs in **(A)** MCF7 cells or **(B)** T47D cells following treatment with scrambled control (SCR) or a second BRCA1 (BRsi#2) specific siRNA.

Supplementary Figure 2 (A) (i) Real time PCR analysis of Jag1 mRNA levels in MCF7 cells following scrambled control (Scr), BRCA1#2 (BRsi#2), DNp63#2 (DNsi#2) or TAp63si (TAsi) specific siRNA treatment. Real time PCR analysis confirmation of knockdowns of **(ii)** BRCA1, **(iii)** DNp63 and **(iv)** TAp63. **(B)** Western blot of HCC BR cells or HCC 1937 cells transiently transfected with empty vector (EV), ΔNp63α, ΔNp63γ, Tap63α or Tap63γ. Blots were probed for Jag1, p63 (4A4) and GAPDH as a loading control.

Supplementary Figure 3 (A) (i) Real time PCR analysis of Notch1 mRNA levels in MCF7 cells following scrambled control (SCR), BRCA1 (BRsi) or DNp63 (DNsi) specific siRNA treatment. **(ii)** ChIP assay in MCF-7 cells showing localisation of RNA polymerase II (RNA Pol II) and BRCA1 (D9 and Ab1) on the Notch1 promoter (Notch 1 pro). One per cent of sonicated lysate prior to immunoprecipitation was used as positive control (input) and IgG pulldowns were used as negative control (IgG1 and IgG2a). **(B)** Real time PCR analysis of a panel of basal, luminal and normal breast cell lines assessed for **(i)** Notch1, **(ii)** Notch2 and **(iii)** Notch 3 mRNA levels. **(C)** Real time PCR analysis of Notch1 and p53 mRNA levels in **(i)** MCF-7 and **(ii)** HCC-BR cells following scrambled control (SCR) and p53 siRNA (p53si).

Supplementary Figure 4 (A) (i) Real time PCR analysis of Notch1 and GATA3 mRNA levels in MCF-7 cells following scrambled control (SCR) or GATA3 (GATA3si) specific siRNA treatment. **(ii)** Luciferase reporter assay of MCF-7 cells pre-treated with either scrambled control (SCR) or GATA3 (GATA3si) specific siRNA treatment followed by transfection with either a Notch1 promoter construct (Notch1 pro) or the pGL3 empty vector control and co-transfected with a Renilla luciferase control construct. Luciferase values were normalised to Renilla and pGL3 and expressed as relative luciferase units (RLU). **(iii)** ChIP assay showing BRCA1 and GATA3 recruitment to the Notch 1 promoter (Notch1 pro) following treatment with either scrambled control (SCR) or GATA3 (G3si) specific siRNA. One per cent of sonicated lysate prior to immunoprecipitation was used as positive control (input) and IgG pulldowns used as negative control (IgG control).

Supplementary Figure 5 (A)(i) Western blot of MCF7 cells stably transfected with Scr or ΔNp63 specific siRNA followed by transient transfection with myc tagged ΔNp63α or TAp63α. Blots were probed for myc tag or GAPDH as a loading control to confirm specificity of the ΔN specific shRNA. **(ii)** Western blots of MCF-7 cells either stably transfected with BRCA1, p63 or Jag1 shRNA or transiently transfected with Notch1, Notch2 and Notch3 siRNA. Western blots were then probed with the relevant antibody to demonstrate efficacy of RNAi. **(iii)** MCF-7 cells were stably transfected with pSuper, BRCA1 #10 and p63 DBD4 shRNA or transiently transfected with scrambled (scr) or Jag1 specific siRNA. Cells were then grown as tumourspheres before being counted and expressed as % mammary forming units relative to pSuper or Scrambled control. **(iv)** Luciferase reporter assay of the same

MCF-7 cells as (A) as well as additional shRNA (BRCA1sh9, ΔNp63sh and Jag1sh) following transfection of a Notch-responsive β-globin reporter construct (pSH2) and an empty vector control (pyLuc) with co-transfection of a Renilla luciferase construct used for transfection control. (**B)** RqPCR of luminal and basal markers in MCF-7 cells treated with scrambled (Scr) control **((i) and (iii))** JAG1#2 or **((ii) and (iv))** ΔNp63#2specific siRNA. β-tubulin mRNA was used for normalisation.

Supplementary Figure 6 (A)(i) Tumoursphere cultures of MCF-7 treated with Scrambled (Scr), Notch1 (Notch1#1), Notch2 (Notch2#1), Notch3 (Notch3#1), a combination of Notch1, 2 and 3 (Notch1,2&3#1). Tumourspheres were counted and expressed as % Mammary Forming Units (MFUs) relative to scrambled control. **(ii)** Aldefluor activity assay of the same MCF-7 cells with % Aldefluor positive cells calculated and shown. **(B)** RqPCR to confirm knockdown of **(i)** Notch1, **(ii)** Notch2 and **(iii)** Notch 3 siRNA in MCF-7 cells following relevant siRNA transfections. **(C)(i)** Tumoursphere cultures of T47D cells treated with Scrambled (Scr), Notch1 (Notch1#1), Notch2 (Notch2#1), Notch3 (Notch3#1), a combination of Notch1, 2 and 3 (Notch1,2&3#1). Tumourspheres were counted and expressed as % Mammary Forming Units (MFUs) relative to control. **(ii)**Aldefluor activity assay of the same T47D cells with % Aldefluor positive cells calculated and shown. **(D)** RqPCR to confirm knockdown of **(i)** Notch1, **(ii)** Notch2 and **(iii)** Notch 3 siRNA in T47D cells following relevant siRNA transfections. **(E)** Flow cytometry of MCF-7 cells either stably transfected with BRCA1, ΔNp63 or Jag1 shRNA or transiently transfected with Notch1, 2 or 3 siRNA and stained for the cell surface receptors CD49f, EpCAM and CD24. Percentage **(i)** stem, **(ii)** basal or **(iii)** luminal progenitor and **(iv)** mature luminal cells are shown. **(F)** RqPCR of lineage and stem cell markers in MCF-7 cells treated with Scrambled (Scr) or **(i)** Notch1#2 (Notch1 #2 si), **(ii)** Notch2 #2(Notch2 #2 si) or **(iii)** Notch3#2 (Notch3#2 si) siRNA.

Supplementary Figure 7 Box plots of relative gene expression from microarray analysis of an in-house dataset **(A – C)** or a publicy available Pawitan dataset **(D – F)** analysed for **(A and D)** Notch receptors and ligands, **(B and E)** markers of differentiation and **(C and F)** stem cell and proliferation markers. Samples are denoted as BRCA1 'sporadic' (grey boxes) and BRCA1 'mutant' (white boxes).

Supplementary Figure 8
(A) Luciferase reporter assay of MCF-7 cells following transfection of a Notch-responsive β-globin reporter construct (pSH2) and an empty vector control (pyLuc) with co-transfection of a Renilla luciferase construct used for transfection control. Cells were pretreated with scrambled or Jag1 (Jag1si) specific siRNA. (**B)** Dose response curve of T47D cells treated with different doses of Tamoxifen (10^{-10} to 10^{-4} M, 72 hours) following treatment with scrambled (SCR) control or JAG1 (JAG1si) specific siRNA. **(C)** Dose response curve of T47D cells treated with different doses of Tamoxifen (10^{-10} to 10^{-4} M, 72 hours) following pre-treatment with Gamma Secretase Inhibitor (GSI, 0.5 and 1μM). **(D)(i)** Luciferase reporter assay of T47D cells following transfection of a Notch-responsive β-globin reporter construct (pSH2) and an empty vector control (pyLuc) with co-transfection of a Renilla luciferase construct used for transfection control. Cells were pretreated with scrambled or Jag1 (Jag1si) specific siRNA. **(ii)** Relative growth of T47D cells 5 days following scrambled (Scr) or Jag1 (Jag1si) specific siRNA.

Mammosphere cultures

Monolayer cells were disaggregated through sequential enzymatic (Trypsin-EDTA) and manual (25G Blunt needle) dissociation to a single cell suspension. This was confirmed microscopically. Cells were plated at 500cells/cm^2 on Ultra-Low attachment Surface plates (Corning Incorporated). These plates, in our experience, results in greater mammosphere forming efficiencies/efficacy compared to "manually" coating plates with polyhema. Cells were then cultured for 7 days in Phenol-Red free DMEM/F12 containing B27 without Vitamin A, EGF, Insulin and Hydrocortisone under normal cell culture conditions. Mammary forming units (MFU) was calculated as the number of mammospheres (greater than 50μm) divided by the total number of cells plates relative to control.

Antibodies

Primary mouse monoclonal antibodies to BRCA1 (AB-1 Oncogene), (D-9), p63 (4A4) (both Santa Cruz Biotechnology, Santa Cruz, CA),cytokeratin 18 (Abcam), p-cadherin (BD transductions), myc tag (Cell Signalling), RNA Polymerase II (Upstate), GAPDH (Biogenesis), and β-tubulin (TUB2.1) (Sigma, Dorset, United Kingdom); primary rabbit polyclonal antibodies to Notch1 (NECD) (Millipore) Notch2,Notch 3 and ERα (HC-20) (Santa Cruz Biotechnology, Santa Cruz, CA); primary goat polyclonal to Jagged-1 (Santa Cruz Biotechnology, Santa Cruz, CA); IgG1 and IgG2a negative controls (Dako) were used.

siRNA oligonucleotide sequences

BRCA1	5′-GCGUGCAGCUGAGAGGCAU-3′
BRCA1 #2	5'- CCATACAGCTTCATAAATA-3'
ΔNp63	5′- GAAGAAAGGACAGCAGCAU -3′
ΔNp63#2	5'- ggA CAg CAg CAT TgA TCA A -3'
Notch1	5′- UGGCGGGAAGUGUGAAGCG -3′
Notch1#2	5'- TGGACAAGATCGATGGCTA-3'
Notch2	5'- GUCUCAGAAGCUAACCUAA -3'
Notch2#2	5'- GGAAGAAGTCTCTGAGTGA-3'
Notch3	5'- CACCUAUAACUGCCAGUGC -3'
Notch3#2	5' – GCCTAGACCTGGTGGACAA-3'
JAG1	5′- CGCCAAAUCCUGUAAGAAU -3′
JAG1#2	5'- ggA CAA ACA AAC Agg ACA A-3'
Scrambled	5′-CCUGGUAGCAGCGAGUCAG-3′
p53	5'- UGUUCCGAGAGCUGAAUGA-3'
TAp63	5' – UUCCUCAGUCCAGAGGUUU-3'
GATA3	5'- AAGCCUAAACGCGAUGGAUAU-3'

RqPCR primer sequences

ΔNp63	F:	5'-gAg TTC TgT TAT CTT CTT Ag-3'
	R:	5'-TgT TCT gCg CgT ggT CTg-3'
TA p63	F:	5'-ATg TCC CAg AgC ACA CAg AC-3'
	R:	5'- CAC ATg ggg TCA CTC Agg TC -3'
Pan p63	F:	5'-gAC Agg AAg gCg gAT gAA gAT Ag-3'
	R:	5'-TgT TTC TgA AgT AAg TgC Tgg TgC-3'
Notch1	F:	5'-CAg gTC AgT ACT gTA CCg Ag-3'

	R:	5'-Tgg CAC TCT ggA AgC ACT gC-3'
Notch2	F:	5'- ACA TCA TCA CAg ACT Tgg TC -3'
	R:	5'- CAT TAT TgA CAg CAg CTg CC -3'
Notch3	F:	5'- Tgg ATg AgT gTC AgC TgC Ag -3'
	R:	5'- Agg TgC AgC TgA AgC CAT Tg -3'
Jagged-1	F:	5'- ggA CAA ACA CCA gCA gAA Ag -3'
	R:	5'- Agg CAA CAA gTA ATg AgA AgA g -3'
Dll-1	F:	5'- CCT ACT gCA CAg AgC CgA TCT -3'
	R:	5'- ACA gCC Tgg ATA gCg gAT ACA C -3'
ERα	F:	5'- TgA TgA TTg gTC TCg TCT gg -3'
	R:	5'- ATT TTC CCT ggT TCC TgT CC -3'
GATA3	F:	5’ – CAG ACC ACC ACA ACC ACA CTC T-3’
	R:	5’ – ggA TgC CTT CCT TCT TCA TAg TCA-3’
SMA	F:	5’ – CTg TTC CAg CCA TTC TTC AT -3’
	R:	5’ – TCA TgA TgC TgT TgT Agg Tgg T -3’
P-cadherin	F:	5'- gCg gAg ACA ggC TAT gAg TC -3'
	R:	5'- AgT CAA ACT gCC CAC ATT CC -3'
β-tubulin	F:	5'- CgC AgA AgA ggA ggA ggA TT -3'
	R:	5'- gAg gAA Agg ggC AgT TgA gT -3'
Muc1	F:	5'-ACA ATT gAC TCT ggC CTT CC-3'
	R:	5'-CAg gTT ATA TCg AgA ggC TgC T-3'
CD61	F:	5’- CgC TAA ATT TgA ggA AgA ACg-3’
	R:	5’- gaAA ggT AgA CgT ggc CTC TTT-3’
CD10	F:	5’- CCg AgA AAA ggT ggA CAA AgA-3’
	R:	5’ – ggA CTg CTg ggc ACT AAA gAA-3’
CD44	F:	5’- TTT gCA TTg CAg TCA ACA gTC-3’
	R:	5’- gTT ACA CCC CAA TCT TCA TgT CCA C-3’
Nanog	F:	5’-CTA AgA ggT ggC AgA AAA ACA-3’
	R:	5’-CTg gTg gTA ggA AgA gTA AAg g-3’
Thy-1	F:	5’-CCC AAT CCC TCA AAC CTT gAg-3’
	R:	5’-gCA Agg ATg ACC CCT CCA gT-3’
FoxM1	F:	5’- Tgg CgA TCT gCg AgA TTT-3’
	R:	5’- CCT CCT CAg CTA gCA gCA CT-3’
CXCL1	F:	5’- TCC gTg gCC ACT gAA CTg-3’
	R:	5’- gTg gCT ATg ACT TCg gTT Tg-3’
CTPS1	F:	5’- ATC CCg Tgg TCg TAg AC-3’
	R:	5’ – Tgg CCA ACA AAC TTC AA-3’
CXCR1	F:	5’ – TgC ATC AgT gTg gAC CgT TA-3’
	R:	5’ – TgT CAT TTC CCA ggA CCT CA – 3’
CXCR4	F:	5’ – gCC AAC gTC AgT gAg gCA gAT g-3’
	R:	5’- gAg gAT gAC TgT ggT CTT gAg g -3’
ABCG2	F:	5`- Cgg gTg ACT CAT CCC AAC AT-3`
	R:	5’ - CAg gAT CTC Agg ATg CgT gC -3’

DNA primers for ChIP assays

J1IER	F:	5'- AAC ATg CAg AgT CCT CTA CCA -3'
	R:	5'- TTA CCT gTT TgA AAA gCA TCC -3'
Notch 1	F:	5`- CCgCTCgAgCgggTgACCgAggAgCgTgTC -3`
	R	5’-CCCAAgCTTgggCTAgCCCAgCggCTTCAC-3’

DNA primers used to generate luciferase reporter constructs
JAG1
F: 5'- CAC TgCCCg CTC gAg Cgg AAC ATg CAg AgT CCT CTA CCA-3'
R: 5'- CAC TgC CCC AAg CTT GGG TTA CCT gTT TgA AAA gCA TCC-3'
Notch1
F: 5'- CCg CTC gAg Cgg gTg ACC gAg gAg CgT gTC-3'
R: 5'- CCC AAg CTT ggg CTAg CCC AgC ggC TTC ACT-3'

DNA primers used to generate shRNA constructs
JAG1 F Forward
5' – gAT CCC C**gA ATg gAg TAC ATC gTA TA**T TCA AgA gA**T ATA CgA TgT ACT CCA TTC** TTT TTA -3'

Reverse
5' –AgT TTA AAA A**gA ATg gAg TAC ATC gTA TA**T CTC TTg AA**T ATA CgA TgT ACT CCA TTC** ggg -3'

BRCA1#9
Forward
5'- gAT CCC C**gA AgC CAg CTC AAg CAA TA**T TCA AgA gA**T ATT gCT TgA gCT ggC TTC** TTT TTA-3'

Reverse
5'- AgC TTA AAA A**gA AgC CAg CTC AAg CAA TA**T CTC TTg AA**T ATT gCT TgA gCT ggC TTC** ggg-3'

DNp63sh
Forward
5' – gAT CCC C**gg ACA gCA gCA TTg ATC AA**T TCA AgA gA**T TgA TCA ATg CTg CTg TCC** TTT TTA -3'

Reverse
5' – AgC TTA AAA A**gg ACA gCA gCA TTg ATC AA**T CTC TTg AA**T TgA TCA ATg CTg CTg TCC** ggg -3'

Analyses of microarray datasets from patient samples:
In the first in-house data set 10 BRCA1 mutant and 10 sporadic breast tumours were profiled using the Almac Diagnostics Breast Cancer DSA Array. Samples were matched for age, stage and clinic-pathological features. Nine of the 10 BRCA1 mutant samples were triple negative (ERα- , PR- and Her2 negative) with one sample Her2 positive but ERα- and PR-negative). All of the sporadic samples were ERα-positive, 9 of the 10 PR-positive and 9 of the 10 Her2 negative.
Clinical information for the Pawitan data set is available through GEO GSE1456. The 159 patient samples were profiled on two array platforms, the Affymetrix Human Genome U133A and U133B, i.e. two expression profiles were available for each patient. The BRCA1 mutation status for each patient was extracted from Oncomine (www.oncomine.com). Considering each platform separately, the data was processed as for the first data set. Where a gene occurred in both platforms, separate U133A and U133B results were reported.
All samples were background-corrected, normalised and transformed using the Affy package, justRMA. The Graphics and Affymetrix packages from R/Bioconductor were used in all analyses (http://www.cran.r-project.org][/http://www.bioconductor.org/).

Supp DataSet1

	Figure 1		
	A(ii)	B (ii)	C (ii)
Notch1	0.012037	0.000695908	0.02167915
Notch2	1.105E-05	0.018701669	0.01691989
Notch3	0.0078714	0.009620459	0.00023382
Jag1	0.0096402	0.032172703	0.04755817
DLL1	0.0366661	0.049205387	0.04954334

	Fig2		
	A(ii)	B (ii)	C (ii)
Con	0.0255811	0.078098226	0.02746059
DSL	0.0074754	0.010264515	0.00455896

	Fig3							
	A (ii)	B (i)	B (ii)		C(ii)		D(i)	
Notch1	0.0018206	0.006821951	DNp63	8.48013E-06	Scr vsBrsi	0.017017	DNp63	6.112E-05
Jag1	0.0051559	0.005740271	TAp63	1.75401E-06	Scr vs p63si	0.0032189	Jag1	0.0001009
			Pan p63	0.000628007				

	Fig4									
pSuper vs	A(i)	A(ii)	B (i)		B (ii)		(iii)		(iv)	
BR	0.0019571	0.005485308	Jag1	0.0017	Jag1	0.0017	p63	0.000571	p63	0.0021719
p63	0.0001971	0.000183195	Muc1	0.0107	Nanog	0.0018	Muc1	0.0009845	Nanog	6.023E-05
Jag1	0.016397	0.000494334	CD61	0.00013216	Thy1	0.0001	CD61	0.0001454	Thy1	0.0092239
			CD10	0.0001	FOXM1	0.0017	CD10	0.0246461	FOXM1	0.0022314
			CD44	0.0086	CXCL1	0.0006	CD44	0.003739	CXCL1	0.0038112
			CXCR1	0.0024	p-cad	0.0001	CXCR1	0.0098392	p-cad	6.928E-05
			CXCR4	0.0392	CTPS1	0.0191	CXCR4	0.0109604	CTPS1	0.0072209
			ABCG2	0.0059			ABCG2	0.0244579		

	Fig5			
scr vs	(A)(i)	(ii)	(iii)	(iv)
Notch1	0.0161862	0.0069629	0.04278237	0.071645598

Notch2	6.976E-05	0.023996597	0.00044084	0.066952082
Notch3	0.0027613	0.012874297	0.00041922	0.033184819
Notch123	2.557E-05	0.028955464	0.00029294	0.004013905
ConvsGSI	0.0009604	0.016966002	0.00679388	0.029553491

B (i)		B (ii)		B (iii)		C(i)	Lif	No Lif	C(ii)	HME1	184A1
Notch1	0.0169913	Notch2	0.00387257	Notch3	0.007092365	Brca1	0.000156937	0.0008511	BRCA1	0.0098118	8.063E-05
CD61	0.0082339	CD61	0.01201802	CD61	0.043373548	Notch1	0.001043354	0.000193	Notch1	0.0209433	0.0158504
CD10	0.0004705	CD10	0.00051589	CD10	0.0006931	Notch2	0.166830331	0.0001866	Notch2	0.0001646	0.0193389
CD44	0.0210248	CD44	0.01290045	CD44	0.009334914	Notch3	0.000179925	0.0001074	Notch3	0.0165945	0.0010521
Nanog	0.0042626	Nanog	0.00168936	Nanog	0.029591909	Notch4	0.00032443	0.0292115	Jag1	0.0374518	0.0430706
CXCL1	0.0193215	CXCL1	0.01617988	CXCL1	0.038618457	Jag1	0.001363351	0.0002047	DLL1	0.022795	0.0220996
CXCR1	0.0126718	CXCR1	0.00341158	CXCR1	0.00519427	Dll-1	0.000123954	0.0002895			
CXCR4	0.0068035	CXCR4	0.02189207	CXCR4	0.00318235	Hes1	0.000201517	0.0001386			
ABCG2	0.018453	ABCG2	0.01671718	ABCG2	0.014257454						

	Fig6								
	A(ii)	A(ii)		B(i)		B(ii)		C(i)	
Notch1	4.62E-05	jag1	0.02170782	Con	0.007076066	Con	0.003584834	ERPro	0.0051293
p-cad	0.2517251	p63	0.48334928	DSL	0.000877602	DSL	0.000540047		
SMA	0.0003888	p-cad	0.19217034						
Eralpha	0.0094336	SMA	0.05723037						
Gata3	0.0178321	Eralpha	0.04461346						
		Gata3	0.03831445						

D(i)		D(ii)	0.5μM	1μM	D(iii)	
-4	0.0003262		7.1681E-15	3.9124E-16	Hes1	0.017087861
-5	0.0001888		0.07064775	0.45769345	Eralpha	0.015283011
-6	0.0366448		0.05672656	0.027920863	GATA3	0.014565179
-7	0.1066501		0.26289392	0.031415642	CXCL1	0.01554938
-8	0.0328033		0.24917777	0.280883595	p-cad	0.002306069
-9	0.1441441		0.21025431	0.470841414	CTPS1	0.011844782
-10	0.4999988		0.39453365	0.002446368	FOXC1	0.027512571

Supp Fig1

	A	B
Notch1	0.0015915	0.004606487
Notch2	0.0009101	0.00343983
Notch3	0.0014006	0.001570553
Jag1	0.0021125	0.003858463
DLL1	0.0043557	0.000139618

Supp Fig2

	A (i)		(ii)	(iii)	(iv)
BR	0.0211249		0.00010424	3.24374E-06	0.007254613
DN	0.0202075				
TA	0.0766293				

Supp Fig3

	A (i)		C(i)	C(ii)
BR	0.0009681	Notch	0.33818903	0.438319953
DN	0.4110687	p53	0.0373859	0.030677264

Supp Fig4

	A(i)		A(ii)
Notch1	0.0062282	Notch	1.4458E-05
Gata3	0.0086639	pGL3	0.2359924

	Supp Fig5									
	A(iii)		(iv)		B(i)		(ii)		(iii)	
BRsh	0.0001968	pSuper vs	BRsh9	0.00293205	Jag1	0.012442	p63	0.0002407	Jag1	0.0001525
DNsh	0.0014298		BRsh10	0.000692356	Muc1	0.0799054	Muc1	0.012987	Nanog	0.0116359
Jag1si	0.0195103		ΔNsh	0.001702251	CD61	0.0008435	CD61	0.0003477	Thy1	0.0002971
			DBD4	0.000658813	CD10	0.0469963	CD10	0.0024262	FOXM1	0.002444
			Jag1sh	0.000518417	CD44	0.0111616	CD44	0.0028677	CXCL1	0.0032342
		Scr vs	Jag1si	3.60294E-06	CXCR1	0.001065	CXCR1	0.0007003	p-cad	0.0105366
					CXCR4	0.0072236	CXCR4	0.0002397	CTPS1	0.0662986
					ABCG2	0.0009039	ABCG2	0.0003634		

(iv)	
p63	0.000154237
Nanog	0.001010746
Thy1	0.020444575
FOXM1	0.004632562
CXCL1	0.036108435
p-cad	0.007636393
CTPS1	0.006430756

	Supp Fig6					
	A(i)	(ii)				
Notch1	0.0313587	0.002288125				
Notch2	0.001088	2.05242E-05				
Notch3	0.0012155	1.22904E-05				
Notch123	0.0003967	0.000366043				
	B(i)		(ii)		(iii)	
Notch1 1	0.0013709	Notch2 1	5.1039E-05	Notch3 1	0.000383734	
Notch123 1	8.888E-05	Notch123 1	3.5371E-06	Notch123 1	0.000596495	
Notch 1 2	0.0003805	Notch2 2	4.8009E-06	Notch3 2	0.000458258	
Notch 123 2	5.265E-05	Notch 123 2	5.6852E-06	Notch123 2	0.000360347	

	C(i)	(ii)
Notch1	0.0016649	0.054780149

Notch2	0.0004798	0.000387192
Notch3	0.0121982	0.013919674
Notch123	0.0001319	0.004457703

	D(i)		(ii)		(iii)
Notch1 1	4.264E-05	Notch2 1	0.00026457	Notch3 1	0.002806317
Notch123 1	2.606E-05	Notch123 1	0.0024273	Notch123 1	0.003412484
Notch 1 2	7.365E-05	Notch2 2	0.0001802	Notch3 2	0.003208815
Notch 123 2	5.104E-05	Notch 123 2	4.5487E-05	Notch123 2	0.003937127

E(i)		(ii)		(iii)		(iv)	
Brsi	0.0034247	Brsi	0.02326871	Brsi	0.007873399	Brsi	0.008160321
Dnsi	0.0366328	Dnsi	0.00350303	Dnsi	0.001994917	Dnsi	0.000371344
Notch1si	0.029906	Notch1si	0.01946277	Notch1si	0.001133336	Notch1si	0.00020787
Notch2si	0.1362839	Notch2si	0.06039243	Notch2si	0.040117849	Notch2si	0.000904315
Notch3si	0.0239324	Notch3si	0.00581415	Notch3si	0.006805585	Notch3si	0.003627949
Jag1si	0.0020696	Jag1si	0.01008637	Jag1si	0.021424072	Jag1si	0.010416929

F(i)		(ii)		(iii)	
Notch1	8.639E-07	Notch2	1.6248E-05	Notch3	3.33895E-07
CD61	4.563E-06	CD61	0.00011405	CD61	3.96632E-06
CD10	0.0040504	CD10	0.00188165	CD10	0.009981394
CD44	0.004618	CD44	8.9587E-07	CD44	4.64413E-07
Nanog	0.0003101	Nanog	0.00010613	Nanog	8.06316E-05
CXCL1	0.0030396	CXCL1	0.01405129	CXCL1	0.000381251
CXCR1	0.0022336	CXCR1	0.00021748	CXCR1	0.003376379
CXCR4	0.0030552	CXCR4	0.00050825	CXCR4	0.005427906
ABCG2	0.0013813	ABCG2	0.00578682	ABCG2	0.000370872

Supp Fig 7

See next sheet

Supp Fig 7

ALMAC		GENE	LOG2 FC	P-VALUE
		NOTCH1	1.0478	3.93E-01
		NOTCH2	0.9679	6.84E-01
		NOTCH3	0.9534	1.90E-01
		NOTCH4	0.9287	7.39E-01
		JAG1	0.9363	1.05E-01
		JAG2	0.9802	8.53E-01
		DLL1	0.9729	5.29E-01
		HES1	0.9549	2.18E-01
		MUC1	0.7920	3.55E-02
	CD61	ITGB3	0.8188	1.47E-02
	CD10	MME	0.9107	4.81E-01
		THY1	0.9391	2.80E-01
	CXCR1	IL8RA	0.9870	7.96E-01
		CXCR4	1.1592	6.84E-03
		ABCG2	0.9269	2.18E-01
		FOXM1	1.1868	8.92E-02
		CXCL1	1.1808	1.05E-01
		CDH3	1.4421	6.84E-03
		CTPS	1.1896	4.87E-04

PAWITAN		GENE	LOG2 FC	P-VALUE
		NOTCH1	1.0030	5.73E-01
		NOTCH1B	1.0077	9.12E-02
		NOTCH2	0.9877	1.51E-01
		NOTCH3	1.0035	9.98E-01
		NOTCH4	0.9949	3.25E-01
		NOTCH4B	1.0048	1.97E-01
		JAG1	0.9672	7.09E-02
		JAG1B	0.9454	7.29E-02
		JAG2	0.9943	7.99E-01
		DLL1B	0.9767	3.67E-02
		HES1	0.9773	2.38E-01
		MUC1	1.0137	2.33E-01
	CD61	ITGB3	0.9966	3.33E-01
	CD10	MME	0.9970	3.08E-01
		THY1	0.9902	9.98E-01
		NANOG	0.9858	2.53E-02
	CXCR1	IL8RA	1.0053	6.22E-02
		CXCR4	1.0139	3.27E-01
		ABCG2	0.9604	2.36E-05
		FOXM1	1.0933	5.32E-05
		CXCL1	1.0038	4.80E-01
		CDH3	1.0082	3.72E-01
		CTPS	1.0389	1.06E-02

	Supp Fig8						
	A	B			C	1µM GSI	0.5µM GSI
Scr vs Jag	3.996E-05		-4	0.0007496		0.001139213	0.0127815
			-5	0.00276809		0.014649737	0.3717151
			-6	0.06087576		0.172335051	0.3058589
			-7	0.01891402		0.278710817	0.2371831
			-8	0.35890024		0.484485342	0.2578886
			-9	0.20025691		0.488457207	0.0773902
			-10	0.04144232		0.28650704	0.1684489
